# Mental Task Evaluation for Hybrid NIRS-EEG Brain-Computer Interfaces

**DOI:** 10.1155/2017/3524208

**Published:** 2017-10-18

**Authors:** Hubert Banville, Rishabh Gupta, Tiago H. Falk

**Affiliations:** Energy, Materials, and Telecommunications, Institut National de la Recherche Scientifique, University of Quebec, Montreal, QC, Canada

## Abstract

Based on recent electroencephalography (EEG) and near-infrared spectroscopy (NIRS) studies that showed that tasks such as motor imagery and mental arithmetic induce specific neural response patterns, we propose a hybrid brain-computer interface (hBCI) paradigm in which EEG and NIRS data are fused to improve binary classification performance. We recorded simultaneous NIRS-EEG data from nine participants performing seven mental tasks (word generation, mental rotation, subtraction, singing and navigation, and motor and face imagery). Classifiers were trained for each possible pair of tasks using (1) EEG features alone, (2) NIRS features alone, and (3) EEG and NIRS features combined, to identify the best task pairs and assess the usefulness of a multimodal approach. The NIRS-EEG approach led to an average increase in peak kappa of 0.03 when using features extracted from one-second windows (equivalent to an increase of 1.5% in classification accuracy for balanced classes). The increase was much stronger (0.20, corresponding to an 10% accuracy increase) when focusing on time windows of high NIRS performance. The EEG and NIRS analyses further unveiled relevant brain regions and important feature types. This work provides a basis for future NIRS-EEG hBCI studies aiming to improve classification performance toward more efficient and flexible BCIs.

## 1. Introduction

A brain-computer interface (BCI) is a communication system between a brain and a computer that bypasses the normal brain output pathways [1]. Such systems can be useful to replace, restore, enhance, supplement, or improve the natural output of the central nervous system [2], and have found applications in clinical as well as nonclinical contexts such as entertainment and education [3]. BCIs rely on the recording of brain activity using imaging modalities like electroencephalography (EEG), magnetoencephalography (MEG), near-infrared spectroscopy (NIRS), functional magnetic resonance imaging (fMRI), and others [4–10]. Although most of today's BCI designs use EEG alone to recognize user intent [11], other modalities offer different information about the underlying brain activity and can therefore complement the information obtained with EEG alone. The hybrid BCI (hBCI) approach thus consists of using more than one modality at a time, including at least one brain modality, but possibly including nonneurophysiological modalities as well [12, 13], to improve on the performance and usability of a unimodal system.

BCIs typically rely on the recognition of one or multiple distinguishable brain activity patterns. The most frequent patterns mentioned in the hBCI literature include event-related desynchronization/synchronization (ERD/ERS) elicited by motor imagery, the P300 event-related potential (ERP), and the steady-state visually evoked potential (SSVEP) [11]. Through their extensive use in the literature, these brain activity patterns have been shown to be highly recognizable when used in BCI designs; however, they may not be optimal for all BCI users. First of all, intersubject variability, a phenomenon that describes how neurophysiological signals can differ significantly from an individual to another, inevitably makes particular tasks better suited to some users than others [11]. Finding the optimal set of mental tasks for a user can thus significantly improve the performance and usability of a BCI. Moreover, users who have suffered a brain injury may lose normal functioning of regions of their brain associated with the above-mentioned patterns. For these users who are often the target of BCI systems, it is thus necessary to use different brain activity patterns that recruit other regions of the brain. Finally, BCI paradigms based on P300 and SSVEP, so-called* reactive BCIs*, rely on external visual stimuli to elicit the necessary brain activity patterns. These external stimuli can induce fatigue in the users when used for extended periods of time [14] and can necessitate additional hardware and software components. Internally triggered mental tasks are more attractive from this perspective as they do not require external stimuli.

To alleviate these problems, a promising approach aims at identifying and validating new brain activity patterns for use in BCI paradigms. Various mental tasks that recruit different parts of the brain, such as mental subtraction and mental rotation, were thus investigated in recent studies using EEG [15–25], NIRS [26–34], transcranial Doppler imaging (TCD) [35, 36], NIRS-TCD [37], and fMRI [38]. The most frequently used mental tasks in these articles were mental subtraction, mental object rotation, various verbal fluency tasks, motor imagery, and auditory imagery. These studies attempted either binary or multiclass classification of mental tasks, usually between tasks or against a resting state, and in most cases aimed at finding the combination of tasks that would yield the highest performance in a BCI context as measured by a classification metric.

Among studies that evaluated four or more different tasks in EEG, combinations of a brain teaser, that is, a task that involves mental work, and a dynamic imagery task were usually found to yield the highest performance. In Sepulveda et al., binary classification of mental singing and mental calculation (either addition or subtraction) was found to be in the top five of the best combinations in four of five subjects, with values above 93% in accuracy [17]. Although aiming to differentiate a mental task from resting state instead of from another mental task, Faradji et al. found that a mental multiplication task could produce a maximal true positive rate above 70% while maintaining a zero false negative rate [18]. Research by Friedrich and colleagues has supported the combination of a brain teaser and a dynamic imagery task as an optimal pair numerous times [21, 22, 25]. Pairs formed from a total of seven different mental tasks were evaluated on nine subjects [21]. Word association, mental subtraction, mental rotation, and motor imagery were identified as the most discriminative tasks, leading to Cohen's *κ* (an interrater agreement metric) values above 0.7. The ERD/ERS patterns evoked by brain teasers and dynamic imagery tasks were found to exhibit different characteristics which could explain why this type of combination is optimal [21]. In addition, in that study the authors characterized the subjective appreciation of each task, finding that while ratings were highly variable and no significant differences between tasks were found, word generation received the best rating and mental subtraction received the worst. Similarly, in a separate study, a combination of mental subtraction and motor imagery was found to produce consistent intersession performance in seven out of nine subjects (kappa higher than 0.6) [22]. Other work by Hortal et al. showed four-class classification of right- and left-hand imagined movement, mental counting, and mental alphabet recitation in two subjects [39]. Although the study did not aim at identifying the best pair of tasks, the trained classifier was better at distinguishing different motor imagery tasks from one another than from other tasks, which could be explained by the centrally focused montage. Finally, in another study, eight subjects were trained to control a 4-class BCI in which mental tasks were selected based on an individual basis [25]. The most frequently selected task combinations included motor imagery, mental rotation, and additional brain teaser and dynamic imagery tasks, leading to average performances varying between 44 and 84% accuracy.

Only two studies were found to look at classifying more than two tasks at once using NIRS. In Herff et al. [31], using the hemodynamic information from the prefrontal cortex it was shown that binary classification accuracies around 60% could be obtained with pairs formed of mental subtraction, word generation, and mental rotation on 10 subjects. A more complete assessment of mental tasks using NIRS was reported in Hwang et al. [5], using a full head coverage. Classification accuracies around 71% were found for combinations of motor imagery, mental multiplication, and mental rotation tasks on seven subjects.

Some studies explored the effect of adding supplementary modalities to the classification of various mental tasks to either improve the number of classifiable tasks or improve the robustness of a precise task. Combinations of NIRS and EEG for recognizing motor imagery or execution tasks have been studied following three different approaches. Fazli et al. used a classifier fusion procedure based on the individual classification of EEG and NIRS data to distinguish left- from right-hand motor execution and imagery [40]. Using this approach, an average 5% increase in accuracy was obtained for 13 out of 14 subjects for the motor imagery task when EEG and NIRS were used simultaneously, yielding an average accuracy of 83.2%. The authors recognized the drawbacks of the long hemodynamic response delay that typically precludes the use of NIRS in practical BCIs. A different approach was explored by Khan et al. to increase the number of input commands in the context of four-direction movement control [41]. While the left and right movements were controlled with motor imagery as measured with EEG, the forward and backward movements were controlled with either counting or mental subtraction tasks measured in NIRS. A binary classification of each task against rest was used. Average classification accuracies of 94.7% and above 80% were obtained for EEG tasks and NIRS tasks, respectively. Finally, in another study, NIRS was used to detect the occurrence of a motor imagery task and trigger its classification as left or right imagery using EEG [42]. This approach led to a low false positive rate of 7% and a true positive rate of 88%.

NIRS has also been combined with peripheral physiological signals such as heart rate, respiration, blood pressure, skin temperature, and electrodermal activity measurements. For example, the impact of environment noise on music imagery detection was assessed using NIRS, physiological signals, and a combination of the two [29]. An average accuracy of 83% over eight participants was obtained when NIRS was used in conjunction with physiological signals, corresponding to a 12% gain from a NIRS-only system. Using a similar methodology, Zimmermann et al. explored the detection of a motor execution task [43]. The authors found that adding physiological signals to NIRS significantly improved classification accuracies by around 9% average in seven subjects. Joining the hemodynamic information obtained from NIRS with the ones obtained from TCD, a modality that measures blood flow velocity in the cerebral main arteries, was also shown to be beneficial in the classification of a mental task. Indeed, this approach helped improve unimodal classification of a verbal fluency task by around 7% average across nine subjects in a separate study in [37].

In light of these results, the purpose of the present article is to gain further ground in the investigation of new BCI control tasks, by adopting a hybrid BCI approach in which NIRS and EEG are simultaneously recorded. We seek to complement previous studies such as [21, 33] by carefully analyzing the functioning of such a hybrid system and assessing whether it can provide a gain in performance over standard unimodal approaches. The rest of this paper is structured as follows.  Section 2 presents the methods used to collect and analyze the data.  Section 3 shows the results of the analysis in terms of spatial patterns, selected features, and classification performance.  Section 4 discusses the results in light of previous related studies. Finally, the conclusions drawn from this work are presented in Section 5. A more comprehensive description of the work can be found in [44].

## 2. Methods

### 2.1. Participants

Twelve participants (5 females, 3 left-handed, mean of 24.6 years old), fluent in English and/or French, took part in our NIRS-EEG study. Participants had to complete three separate recording sessions of two to three hours, inside a period of three to five weeks. Participants declared having no history of neurological disorders and had no previous experience with BCIs. A monetary compensation was given after each completed session. Each participant agreed with the terms and conditions of the study, which was approved by the university ethics committee. Two participants were rejected because their data contained a high number of artifacts; a third participant was rejected because they did not complete the three required sessions. Therefore, the data from a total of nine participants was used in this study.

### 2.2. Mental Tasks

Participants were asked to perform seven different types of mental tasks that are believed to elicit specific neural response patterns, based on previous studies using EEG and NIRS [21, 33].

*Mental Rotation (ROT)*. Two 3-dimensional L-shaped figures, either identical or mirrored, but in each case in a different state of rotation, were presented to the participants. Participants had to imagine rotating one of the two figures in order to find if they were the same or if they were mirrored images.

*Word Generation (WORD).* Participants had to generate as many words as possible starting with a randomly chosen letter presented on the screen. Words in either English or French, depending on the participant's chosen language, were requested.

*Mental Subtraction (SUB)*. Participants had to perform successive subtractions of two 1- or 2-digit numbers to a 3-digit number (e.g., 214 − 9 = 205 and 205 − 13 = 192).

*Mental Singing (SING)*. Participants had to imagine singing a song that they chose beforehand, if possible with lyrics, while focusing on the emotional response it elicits.

*Mental Navigation (NAV)*. Participants had to imagine walking from one point to another in their current or a previous home, while focusing on their spatial orientation (e.g., walking from their bedroom to the refrigerator).

*Motor Imagery (MI).* Participants had to imagine performing a finger tapping task with their right hand.

*Face Imagery (FACE). *Participants had to imagine the face of a friend, as recalled from a picture they were asked to bring to the recording session and memorize.

Following Friedrich et al.'s description of task types [21], we classify mental rotation, word generation, and mental subtraction as brain teasers, since they require problem-solving skills; mental singing, mental navigation, and motor imagery as dynamic imagery tasks; and face imagery as a static imagery task.

Before the recording started, participants were first guided through each task and asked to complete them in an overt manner. This step was used to make sure each participant performed the tasks appropriately and in the same manner. For example, for a mental rotation task, participants had to say aloud if the two figures were the “same” or “mirrored,” for a mental subtraction task they had to give the experimenter their final answer, and so on. Participants were then asked to repeat the same tasks but in a covert manner, as they were then asked to during the experiment.

### 2.3. Experimental Paradigm

**Figure 1:**
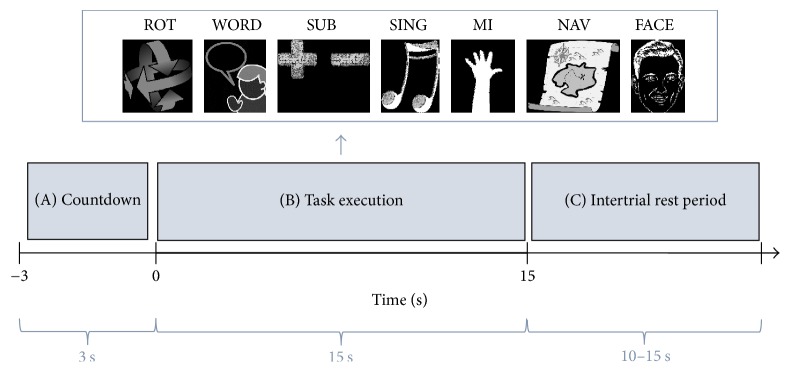
Diagram of a trial of the experimental paradigm. A trial is composed of (A) a 3 s countdown period in which participants are instructed about the coming mental task, (B) an imagery period where they execute the given task for 15 s, and (C) a randomized 10 to 15 s rest period.

The experimental paradigm for a single session of our study is summarized in Figure 1. A session consisted of four subsessions in which each mental task type was randomly repeated four times, yielding a total of 28 task completions per subsession. Participants also had to complete a subjective evaluation questionnaire between the second and third subsession of each session. Each subsession started and finished with a 30 s baseline period in which participants were asked to remain in a neutral mental state and fixate the cross at the center of the screen. Before each trial, a 3 s countdown screen indicated the task to be performed next using the associated pictogram as shown in Figure 1. Once the countdown was over, participants had to execute the required mental task for a period of 15 seconds. Instructions were given to carry out the tasks as many times as possible and to start again tasks such as rotation, subtraction, and navigation if completed before the end of the 15 s period. Each trial was then followed by a rest period of random duration between 10 and 15 s, sampled from a uniform distribution. This randomization keeps the participants from expecting the exact start of the next trial and avoids the synchronization of systemic processes in NIRS with the paradigm. Once a subsession was over, participants were allowed to take as much time as desired to lightly stretch, drink, or eat a snack before resuming the experiment. The stimuli and questionnaire were both implemented using the Presentation software package (Neural Behavioral Systems, USA).

### 2.4. Data Collection

#### 2.4.1. EEG and Physiological Signals

**Figure 2:**
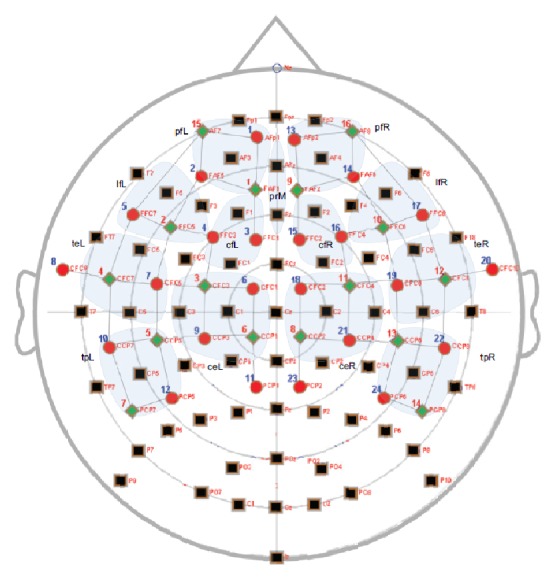
EEG and NIRS topology used in this study (adapted from [59]). EEG electrodes (black rectangles), NIRS detectors (red circles), and NIRS sources (green diamonds) were placed following the 10-5 system. NIRS channels are represented by dark straight lines connecting the sources to the detectors. Brain regions used to compute artificial NIRS channels are grouped in light blue.

EEG data was recorded using an ActiveTwo system (Biosemi B.V., Amsterdam, The Netherlands) with 62 probes (plus two mastoids and CMS and DRL electrodes) and four EOG electrodes, digitized at 512 Hz. No online filtering was applied. A standard 10-10 system was used for electrode placement, but without AF7 and AF8, whose holes were used for NIRS probes instead (see Figure 2).

#### 2.4.2. NIRS

NIRS data was recorded using a NIRScout system (NIRx Medical Technologies, Los Angeles, USA), with 16 sources (wavelengths of 760 and 850 nm) and 24 detectors. Optodes were placed together with EEG electrodes on the same cap, as shown in Figure 2. The frontal, central, temporal, and parietal lobes were targeted by the used montage, following the extended 10-5 system. Coverage was not extended to the occipital lobe due to the low quality of NIRS signals in this region and because of the restricted number of available optodes. Source-detector pairs separated by approximately 3 cm were used as channels, giving a total of 60 NIRS channels, each sampled at 4.46 Hz. Amplifier gains were adjusted following an automatic calibration procedure handled by the NIRStar recording software.

#### 2.4.3. Questionnaire and Subjective Ratings

At each recording session, participants were asked to fill out a questionnaire reporting their appreciation of the tasks. The questions were based on the first part of the widely used NASA Task Load Index (TLX) test [45], with the French version by Cegarra and Morgado [46]. Additionally, participants were asked to rank the mental tasks in order of preference.  Table 1 shows the various items that were measured.

### 2.5. EEG Analysis

#### 2.5.1. Preprocessing

The raw EEG data was preprocessed using EEGLAB [47]. The data was referenced to Cz and then downsampled to 256 Hz before filtering with a bandpass (0.5–100 Hz) and notch filters (60 Hz). Bad channels were visually assessed and removed if they were constantly bad across trials of the same session. Epochs of 25 seconds with 5 seconds of baseline before and after task execution were extracted for each trial, as well as for rests periods at the beginning and end of each subsession. Epochs contaminated by strong movement and physiological artifacts were visually identified and rejected. On average, 6.1% of the epochs were rejected, yielding an average of 315.4 valid epochs per subject (out of a possible 336). Further signal cleaning was performed by applying a semiautomatic method based on independent component analysis (ICA, with the Infomax algorithm [48]) to detect and subtract remaining artifacts and noise components [49]. Bad channels were reinterpolated and all channels were rereferenced to the average of all electrodes. Finally, baseline correction was applied to each epoch using the 300 ms period before the beginning of the tasks.

#### 2.5.2. Descriptive Analysis

ERD is a phenomenon occurring when idle parts of the brain become active following some event or stimulus. A specific rhythmical activity can then be measured, such as *μ* and *β* waves over the primary motor cortex in motor execution and imagery tasks [50]. Similarly, ERS occurs when this activity ceases and the recruited brain regions return to an idle state. The intertrial variance method proposed in [51] was used to compute the ERD/ERS values for each type of mental task and baseline over all three sessions of a participant.

#### 2.5.3. Feature Extraction

Previous EEG studies mainly used band powers and Common Spatial Patterns-based (CSP) features to describe the neural activity patterns induced by mental tasks such as motor imagery [11]. In our case, since we are especially interested in the interpretation of the extracted features, and given the explicit link between band powers and measurements of ERD/ERS [52], we focused our classification analysis on classical power bands. Each trial was subdivided in nonoverlapping time windows of one second. Log-power features were then extracted in the following seven frequency bands with a Fast Fourier Transform: *θ* (4–8 Hz), *α*_low_ (8–10 Hz), *α*_high_ (10–12 Hz), *β*_low_ (12–21 Hz), *β*_high_ (21–30 Hz), *θ* to *β* (4–30 Hz), and total spectrum (0.1–100 Hz). Individual features for the *δ* (0–4 Hz) and *γ* (30–80 Hz) bands were not extracted in order to reduce the impact of ocular and muscle artifacts [20, 21, 24]. Moreover, the following ratios of band powers were computed: *α*_total_/*β*_total_ and *θ*/*β*_total_. This yielded a total of 620 features per window, which were finally log-transformed.

### 2.6. NIRS Analysis

#### 2.6.1. Preprocessing

NIRS was preprocessed using the open source toolbox HOMER2 [53]. First, raw light intensities were converted to optical densities (OD) by computing the negative logarithm of the normalized intensities (using the average value of each channel over the entire recording). Second, channels with a low signal-to-noise ratio were identified by correlating the bandpass filtered OD (between 0.8 and 1.2 Hz) of channels S15-D1 and S15-D2 on the left and channels S16-D13 and S16-D14 on the right, with each left side or right side channel, respectively. These 4 frontal channels being the shortest in the used montage, and usually being clear of any hair, are expected to carry a clear cardiac pulse in the 45 to 100 BPM range, which corresponds to standard resting heart rate frequencies. Channels which significantly correlate with these short-distance channels in this frequency range (*p* value below 0.05) are thus expected to be of good quality, since they carry physiological information. Channels were rejected per session if the aggregate of their Pearson's correlation *p* value across all epochs was not significant. Third, the remaining channels' OD was bandpass-filtered between 0.01 and 0.30 Hz [10] and converted to the concentration changes of oxygenated, deoxygenated, and total hemoglobin (Δ[HbO], Δ[HbR], and Δ[HbT]) using the Modified Beer-Lambert Law [54]. Finally, 13 artificial channels were computed by averaging the amplitude of neighboring channels in the prefrontal, lateral-frontal, centrofrontal, temporoparietal, and central regions (as shown in Figure 2). These steps produced 73 channels, each one measuring Δ[HbO], Δ[HbR], and Δ[HbT], giving a total of 219 measurement channels for NIRS.

#### 2.6.2. Descriptive Analysis

The average Δ[HbO], Δ[HbR], and Δ[HbT] responses over sessions and participants were computed for each task to reveal spatiotemporal patterns of hemodynamic activation.

#### 2.6.3. Feature Extraction

Classification of NIRS data is often based on simple features such as Δ[HbO], Δ[HbR], and Δ[HbT] averaged over time, their slope, or even the averaged raw light intensity values [11]. In this study, the average chromophore concentrations of each channel were extracted using the same windows as for EEG feature extraction. Features derived from NIRS channels that were rejected during preprocessing were set to 0. This yielded a maximum of 219 NIRS features.

### 2.7. Classification of EEG, NIRS, and EEG-NIRS

Each previously extracted feature set was then used to train a binary classification model over a pair of tasks. First, the training procedure was applied to the EEG and NIRS datasets separately to assess the individual performance of each modality. Then, the same procedure was applied on a merged dataset combining features from both EEG and NIRS to assess the impact of multimodal information on classification performance.

To avoid the overfitting problems linked to high-dimensional datasets, especially in cases where only a few training cases are available, feature selection procedures are required to select the most informative features prior to or during classifier training. In this study, we used a linear kernel Support Vector Machine (SVM) classifier combined with a sparsity-inducing *l*_1_ penalty term to control the number of features used in the model. As opposed to filter feature selection methods, this embedded procedure allows the efficient discovery of interdependent features, while also making better use of the available data by avoiding an additional partitioning [55]. Combined with the robustness of SVMs in high-dimensional spaces [56], this embedded feature selection-classification procedure is designed to minimize the detrimental effects of our high-dimensional dataset with few examples.

A default value of 2.0 was used for the hyperparameter *C* that controls the balance between the data-dependent loss and the *l*_1_ penalization of the weights. To account for the varying number of data points in each classification task, *C* was divided by the number of examples in the training set, yielding a maximal value of 0.023 when all 96 epochs were conserved.

A delay between the cue to execute a mental task and its actual execution is expected; moreover, participants are likely to get tired and stop executing the task before the end of the 15 s epoch. Therefore, to reveal the dynamics of mental task execution, each time window was analyzed independently; that is, a new classifier was trained for each time window (e.g., 0-1 s and 1-2 s). This yields a series of performance estimates that show how classification evolves as the task is executed. The subject-specific classifiers were trained using a 10 times 10-fold stratified cross-validation procedure. Nine partitions were used for training, and the remaining one was used for validation. Partitions were created so that relative class frequencies were similar in each fold. The data was then randomly shuffled and the partitioning procedure repeated for another nine times. This yielded a total of 100 estimates per classification task. For each division of training and validation sets, each feature was then individually *Z*-score-normalized based on the mean and standard deviation of the training set. Finally, the classifier was retrained on all 10 folds to obtain the final weights *w*.

Since our study is targeted toward the eventual conception of a BCI, we focus the bulk of our analysis on models trained with features extracted on windows of one second. This allows a very short delay between the realization of the mental task and the output of the system. Moreover, using one-second windows allows a fine-grained analysis of the temporal evolution of selected features and of classification performance, which can uncover interesting physiological insights.

The performance of the classifiers was evaluated using Cohen's *κ* as in [21], which measures the agreement between two raters who classify *N* examples into mutually exclusive categories. This is useful here since an unequal number of repetitions were kept for each type of tasks, leading to an unbalanced number of examples in the dataset. In the case of a perfectly balanced problem, *κ* values of 0, 0.4, and 1 are equivalent to 50%, 70%, and 100% accuracy, respectively.

To identify the best pairs of tasks for each modality configuration, a procedure that takes into account both the average and the variance of the *κ* sample across participants was used. A two-tailed *t*-test was used to compare the *κ* values obtained with each pair of tasks to 0.4. Following this test, the resulting 21 *t*-statistics were ranked: the larger the *t*-statistic, the greater the performance of a pair. Additionally, to assess the impact of adding NIRS to EEG features, two-tailed paired *t*-tests were used to compare the *κ* values obtained for each pair of tasks of the EEG-only and NIRS-EEG cases. The Holm-Bonferroni method was used to correct for multiple comparisons [57].

The best features can also be analyzed using the SVM weights of these models as a feature ranking metric [58]. Since the features are normalized before training, the weights of the linear model effectively represent the importance of each feature. A feature with a high absolute weight can thus be thought of as being more important than one with a weight closer to 0. In the present study, the absolute values of the weights of a particular feature were averaged and then ranked against the other features.

## 3. Results

### 3.1. Descriptive Analysis

#### 3.1.1. EEG

**Figure 3:**
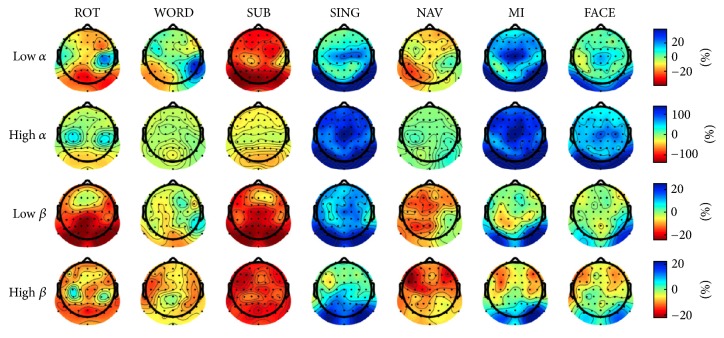
ERD/ERS maps for each task in the low *α*, high *α*, low *β*, and high *β* bands. ERD/ERS values are computed using the intertrial variance method [51] over the time window spanning 0.5 to two seconds after stimulus onset, using a baseline of −2 to 0 seconds before trial onset. Blue represents ERS while red represents ERD. Note that color ranges differ between power bands.

The ERD/ERS maps for each task in the low *α*, high *α*, low *β*, and high *β* bands are shown in Figure 3. In mental rotation, high amplitude ERD patterns were measured in the occipital region for all four bands. A pattern of weak ERS was observed over the two motor cortices in all bands except low *β*. Word generation produced ERS patterns over the left temporal lobe in the low *α* and high *α* bands. Patterns elicited by mental subtraction showed consistently high ERD in the occipital lobe, especially around PO7 and PO8 (both among the most important features identified in Table 3). In mental singing, ERD patterns over the left hemisphere in the low and high *β* bands were observed, as well as predominant all-band ERS in the occipital region. Mental navigation led to left hemispheric ERD in the low *α* and low *β* bands, as well as left and right prefrontal ERD in the high *β* band. In motor imagery, left hemispheric ERD patterns were dominant in the first three bands, except for a bilateral high *α* ERD pattern in the prefrontal lobe, similar to the one observed for NAV but of lower intensity. Finally, face imagery led to ERD patterns in the frontal (again, similar to the ones observed for NAV and MI in the high *β* band) and temporal lobes, as well as high *α* power in the occipital lobes.

#### 3.1.2. NIRS

**Figure 4:**
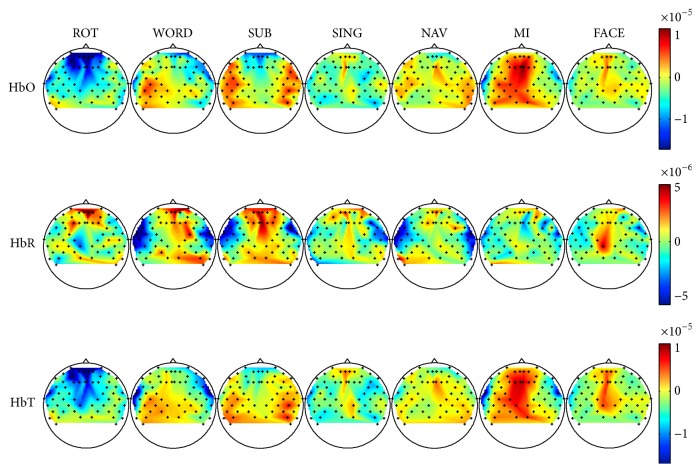
Average HbO, HbR, and HbT maps for each task. Reported HbO, HbR, and HbT values are normalized to their baseline values and averaged across the time window spanning 10 to 15 seconds after stimulus onset. Red represents an increase in concentration of the chromophore, while blue represents a decrease (in mmol/L).

Figure 4 shows the average Δ[HbO], Δ[HbR], and Δ[HbT] topographical maps for each task during the time window spanning 10 to 15 seconds after stimulus onset. Mental rotation led to a decrease in Δ[HbO] and Δ[HbT] over the frontal lobe, as well as an increase in Δ[HbR] over the prefrontal lobe. In turn, word generation yielded an increase in Δ[HbO] over the left temporal lobe and a decrease in Δ[HbR] over the left and right temporal lobes. This was accompanied by a widespread increase in Δ[HbT] over the posterior left hemisphere. In mental subtraction, an increase in Δ[HbO] and Δ[HbT] over both the right and left temporal lobes, as well as a decrease in the prefrontal region, was observed. Oppositely, Δ[HbR] increased over the midline but decreased in both temporal lobes. Mental singing provoked a subtle decrease in Δ[HbR] over the temporal lobes, similar to WORD and SUB. The mental navigation task led to decreased Δ[HbR] levels over both temporal lobes and increased Δ[HbO] levels in the same regions. A large increase in Δ[HbO] and Δ[HbT] was observed over the left hemisphere for the motor imagery task, without any patterns of similar amplitude in Δ[HbR]. Finally, face imagery led to an increase in the levels of all chromophores on the midline. A consistently high Δ[HbR] pattern over the right centroparietal region can also be seen in most tasks.

### 3.2. Subject-Wise Mental Task Classification

In this section, the results of EEG-only and NIRS-only classification are described, followed by the results of NIRS-EEG fusion classification. Specifically, the peak classification performance across subjects and across task pairs, the evolution of kappa across time, and the feature selection results are described.

#### 3.2.1. EEG Only

The peak classification performance of classifiers trained on one-second window EEG features, for each subject and pair of tasks, is shown in Table 2. The peak classification performance is defined as the highest *kappa* obtained across the 15 seconds of task execution. All subjects achieved satisfactory or high performance for most task pairs and with an average *κ* greater than 0.4. High performance was achieved for at least three different task pairs in all subjects but S04 and S09, and three subjects (S10, S03, and S06) showed an average peak *κ* greater than 0.7.

Twelve pairs of tasks yielded *κ* values significantly greater than 0.4. The seven best performing task pairs were combinations of a brain teaser and an imagery task: either ROT or SUB with SING, FACE, MI, or NAV. For instance, the ROT-MI pair had an average *κ* of 0.83. However, pairs of dynamic and static imagery tasks, including SING, FACE, NAV, and MI, showed a consistently lower *κ*; for example, SING-FACE led to the lowest average *κ* of 0.4.

**Figure 5:**
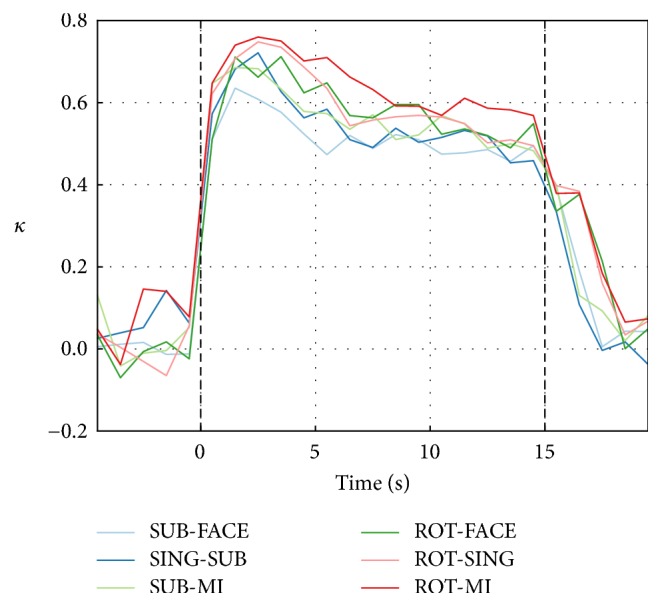
EEG-only classification *κ* over nonoverlapping one-second windows for the six best task pairs. The classification *κ* obtained with one-second windows was averaged over participants for each task pair. Each point is aligned with the middle of the window from which the features were extracted. Baseline *κ* values were not significantly greater than 0 (one-tailed *t*-tests).

The average classification *κ* over subjects, computed across one-second windows, is shown in Figure 5 for the six best task pairs. *κ* values increase quickly after stimulus onset and reach a peak around four seconds later, as seen also in Table 2. The performance then decreases gradually over the remaining 11 seconds of the trial and returns to chance level three seconds after the end of the task.

The *l*_1_-SVM algorithm selected a minimum of zero and a maximum of 12 EEG features, with a median of seven features. The case where zero features are selected simply means that all the SVM weights are zero, so that the classifier always outputs the same decision, dependent on its bias. During the pre- and postepoch baseline, only around one or two features are selected. This number increases quickly after stimulus onset and remains stable across the 15 seconds of the epoch. It is interesting to note that S04, who obtained the lowest average peak *κ*, also consistently had the lowest number of selected features.

**Figure 6:**
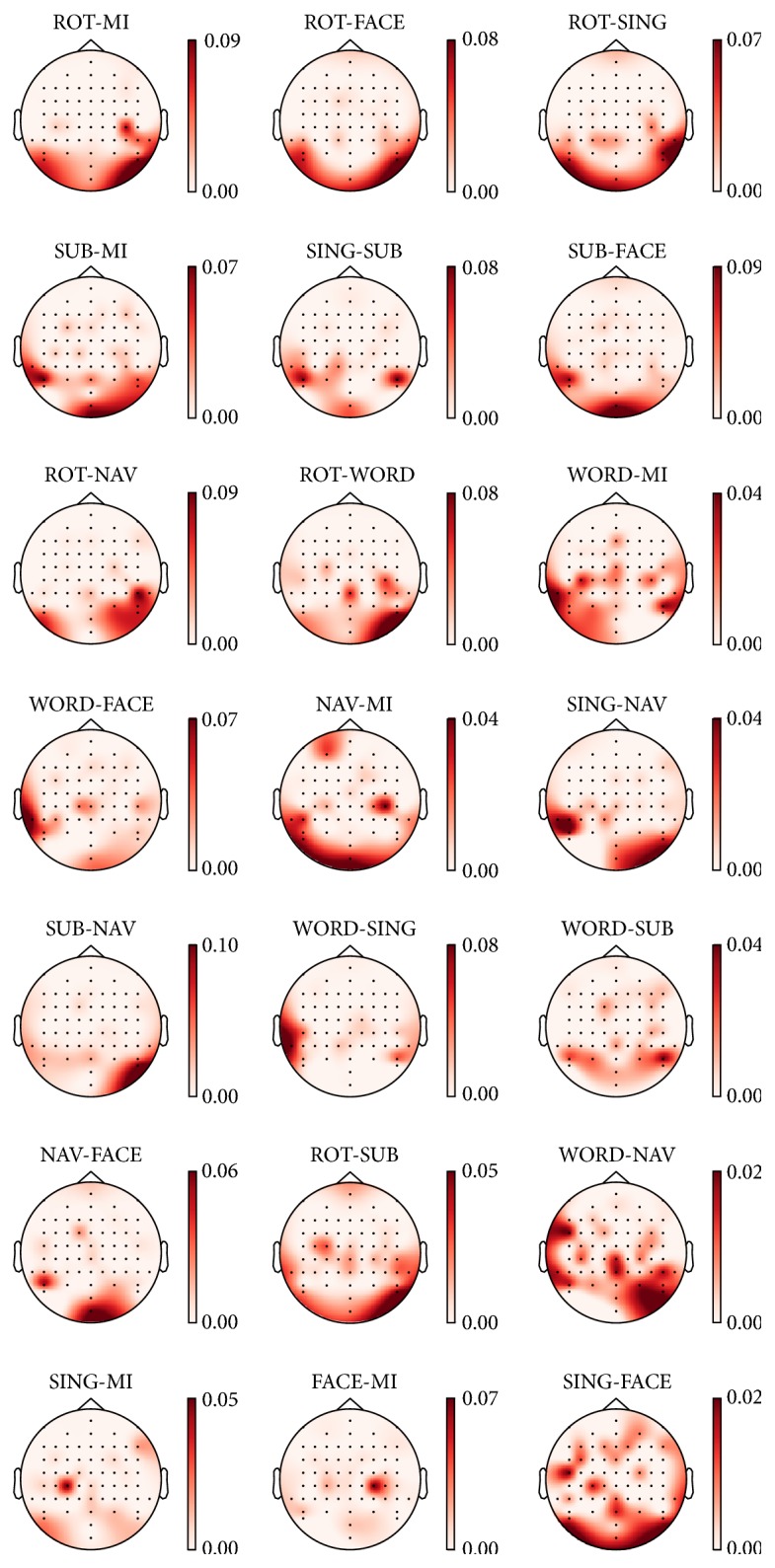
Topographical maps of the average absolute value of the SVM weights, when trained on EEG features only. Darker regions are those that are more important when classifying each task pair. Note that distinct color ranges are used for each map. This figure uses the models trained on the one-second window occurring three to four seconds after stimulus onset, which corresponds to the average peak time for EEG classification (see Table 2). The pairs are plotted in descending order of average *κ* (left to right and top to bottom) as presented in Table 2.

The five most important features following the approach described in Section 2.7 are listed in Table 3 for the five task pairs that produced the highest performance. Note that *α*- and *β*-related features showed strong importance, while *θ* and wide-band features were typically not highly ranked. Most of these features were extracted from electrodes located in the parietal and occipital regions. To support this observation, the average absolute values of the SVM weights are visualized on a topographical map of the head to show the importance of each channel according to the trained classifier (see Figure 6). In almost every case, channels in the occipital and parietal regions showed the highest feature importance. High performance pairs (first three rows in Figure 6) all displayed maximum feature importance for channels at the back of the head. A pattern of high importance for channels PO7 and PO8 can be noticed in many pairs (i.e., ROT-FACE, SUB-FACE, ROT-NAV, SING-SUB, and WORD-NAV).

#### 3.2.2. NIRS Only

The peak classification performance of classifiers trained on one-second window NIRS features, for each subject and pair of tasks, is shown in Table 4. The best overall *κ* is again obtained by S10, with 13 different pairs of tasks reaching high performance. S07, S04, and S11, respectively, ranked fifth, last, and seventh in the EEG-only analysis; all achieved an average *κ* above 0.50. On the other hand, S03, who ranked second in the EEG-only analysis, was ranked second to last in the NIRS-only case. The average peak time across task pairs is 11.5 seconds and again shows high variability.

Fifteen pairs of tasks were classified with average *κ* above 0.4, but only three yielded values significantly greater than 0.4. The best performing pairs were mostly a combination of a brain teaser and an imagery task (ROT-MI, ROT-SING, ROT-FACE, and SUB-MI), with the exception of ROT-WORD. Pairs combining two imagery tasks, such as SING-MI, NAV-FACE, FACE-MI, and SING-FACE, were constantly classified with the lowest average *κ*. Similar to the EEG-only case, ROT-MI achieved top-3 performance, while FACE-MI and SING-FACE achieved the lowest. ROT and SUB tasks were the most useful overall when paired with passive imagery tasks, whereas pairs of imagery tasks were all under the 0.4 *κ* threshold.

**Figure 7:**
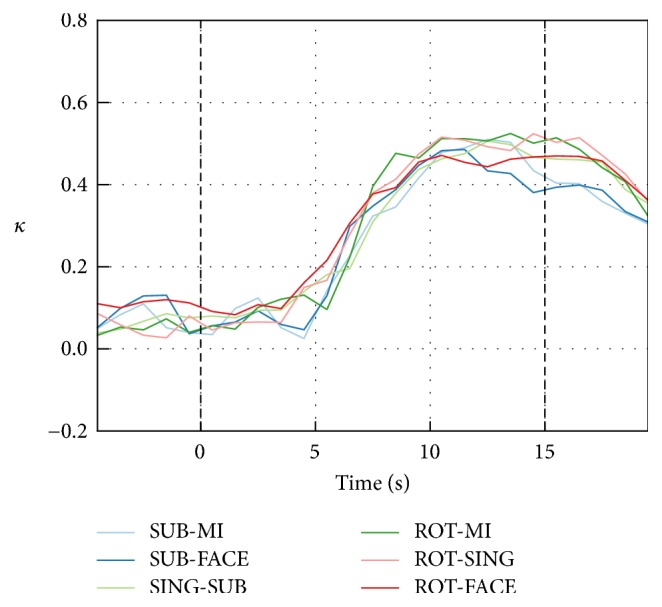
NIRS-only classification *κ* over nonoverlapping one-second windows for the six best task pairs. The classification *κ* obtained with one-second windows was averaged over participants for each task pair. Each point is aligned with the middle of the window from which the features were extracted. Baseline *κ* values were not significantly greater than 0 (one-tailed *t*-tests).

The average classification *κ* over subjects, computed across one-second windows, is shown in Figure 7 for the six best task pairs. The *κ* values of the best task pairs oscillated around *κ* = 0 during the baseline and the first five seconds of the trial. *κ* values then started rising approximately five seconds after stimulus onset and reached a plateau around six seconds later, which is consistent with the expected hemodynamic response delay [8]. As shown in Table 4, this leads to an average peak time of 11.5 s. Performance decreased after approximately eight seconds and did not return to preepoch baseline levels in the five seconds after the end of the trial.

When applied to NIRS features, the *l*_1_-SVM algorithm selected a minimum of zero and a maximum of 11 features, with a median of three features. Only around one feature was selected during the preepoch baseline and up to five seconds after stimulus onset, after which this number increased and remained more stable until the end of the task and the postepoch baseline. Subject 10, who obtained the highest average peak *κ*, also consistently had the highest number of selected NIRS features.

**Figure 8:**
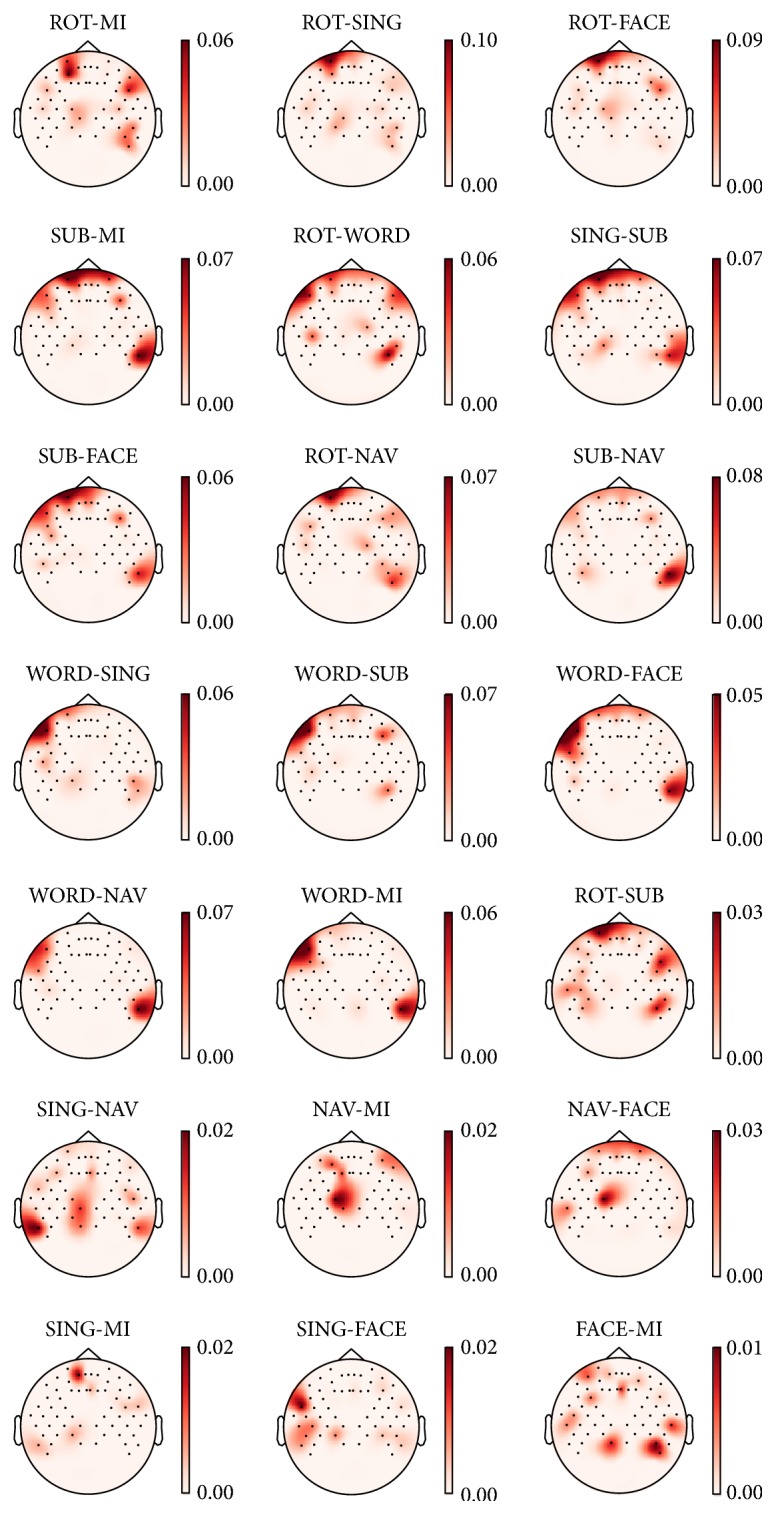
Topographical maps of the average absolute value of the SVM weights, when trained on NIRS features only. Darker regions are those that are more important when classifying each task pair. Note that distinct color ranges are used for each map. This figure uses the models trained on the one-second window occurring 11 to 12 seconds after stimulus onset, which corresponds to the average peak time for NIRS classification (see Table 4). The pairs are plotted in descending order of average *κ* (from left to right and top to bottom) as presented in Table 4.

The five most important features are listed in Table 3 for the five best task pairs. Sources 13 (CCP6) and 14 (PCP8) over the right parietal cortex and sources 15 (AF7) and 16 (AF8) over the right and left prefrontal cortex led to the highest ranking scores. Δ[HbR] and Δ[HbO] features generally showed more importance than Δ[HbT] features. To further identify regions of interest, the average absolute values of the SVM weights are visualized on a topographical map of the head in Figure 8. Channels in the prefrontal region showed consistently high importance for pairs including ROT and SUB tasks, whereas channels in the left prefrontal region showed consistent high feature importance in pairs that include the WORD task.

#### 3.2.3. Fusion of Signals

The peak classification *κ* values obtained for models trained on concatenated EEG and NIRS features are shown in Table 5. The three best pairs of tasks remained the same as for EEG-only classification (ROT-FACE, ROT-MI, and ROT-SING), as did the three worst pairs (SING-MI, FACE-MI, and SING-FACE). Again, most of the highest ranked task pairs were a combination of a brain teaser and an imagery task. Moreover, the task-wise *κ* values exhibited the same top-7 ranking as for EEG-only classification. Subject-wise, the four best participants (S10, S03, S07, and S06) were the same as for EEG-only classification. The other five subjects saw their ranking change by one or two positions. On the other hand, the peak time increased to an average of 6.4 seconds.

**Figure 9:**
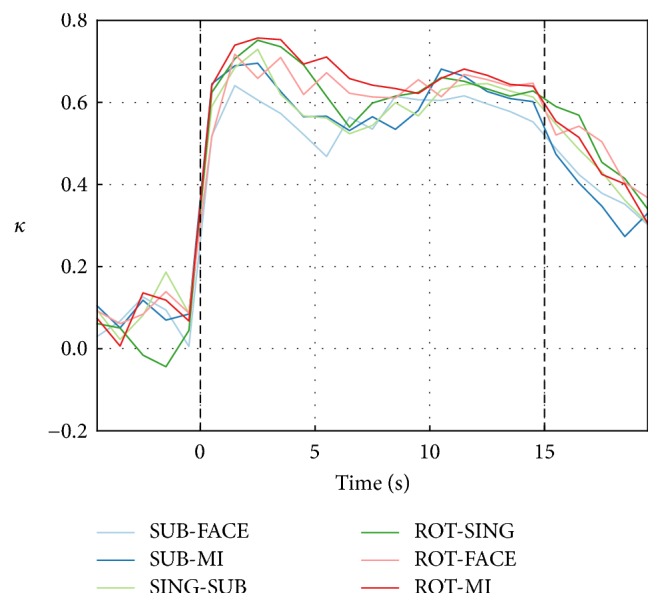
EEG and NIRS classification *κ* over nonoverlapping one-second windows for the six best task pairs. The classification *κ* obtained with one-second windows was averaged over participants for each task pair. Each point is aligned with the middle of the window from which the features were extracted. Baseline *κ* values were not significantly greater than 0 (one-tailed* t*-tests).

The average classification *κ* over subjects, computed across one-second windows, is shown in Figure 9 for the six best task pairs. Two peaks can be observed: first, at three seconds after trial onset, corresponding to the peak *κ* obtained with EEG features alone; and second, at around 11 seconds after trial onset, corresponding to the peak *κ* obtained with NIRS features alone. More specifically, when comparing these values to the ones obtained with EEG alone, we see that the improvement seems small in the first eight seconds of the task (as reported in Table 5) but is more noticeable in the last five seconds of the task, where more than one NIRS feature was originally selected.

To get a better idea of how feature fusion of EEG and NIRS impacted classification performance, Table 6 shows the increase in peak *κ* obtained by adding NIRS to EEG features, evaluated at the time windows where NIRS classification yielded the highest performance (as reported in Table 4). The average increase was of 0.20, while the highest increase was of 0.84, achieved by S10 on task pair SING-SUB. Average performance gains were the highest for S04 (Δ*κ* = 0.40), S11 (Δ*κ* = 0.30), and S07 (Δ*κ* = 0.28).

The seven best task pairs included either WORD or NAV (or both), while task pairs that included FACE or MI benefited less from fusion on average.

The *l*_1_-SVM algorithm selected a minimum of zero and a maximum of 15 features with a median of seven features when trained on the concatenated EEG-NIRS dataset. The behavior is almost identical to that exhibited by EEG-only models, until around 11 seconds after stimulus onset, where a slight increase in the number of selected features is observed. This effect is particularly apparent for S04, who benefited the most from the fusion.

**Figure 10:**
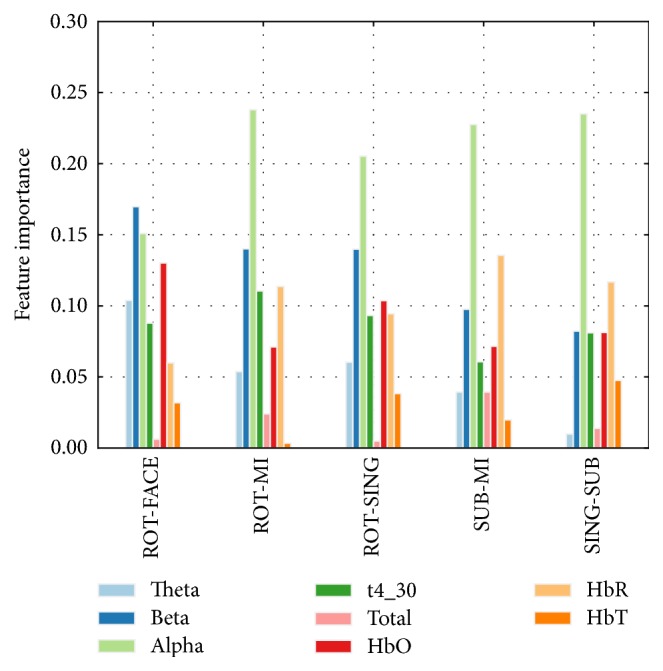
Importance of each EEG and NIRS feature subtype: EEG band powers (*θ*, *α*, *β*, 4–30 Hz, and total spectrum (0.1–100 Hz)) and chromophore types (HbO, HbR, and HbT). The histogram is based on the models trained on the one-second window occurring 11 to 12 seconds after stimulus onset, which corresponds to the average peak time for NIRS classification (see Table 4).

The five most important features per task are listed in Table 3 for the five best task pairs. Most of the previously chosen features are selected again.  Figure 10 shows the relative importance of each subtype of feature (band power and chromophore type) for the same six task pairs. We see that *α*-related features were generally the most important, followed by *β*-related features. On the other hand, the *θ*, 4–30 Hz, and 0.1–100 Hz bands were usually less important. In NIRS, HbR-related features showed the strongest importance (while still inferior to *α*-related features), followed closely by HbO-related features. HbT did not show much importance. In some participants (S03, S07, and S11), HbR-related features were consistently more important than HbO-related ones, while it was the opposite for S04 and S09 (results not shown).

### 3.3. Questionnaire

**Figure 11:**
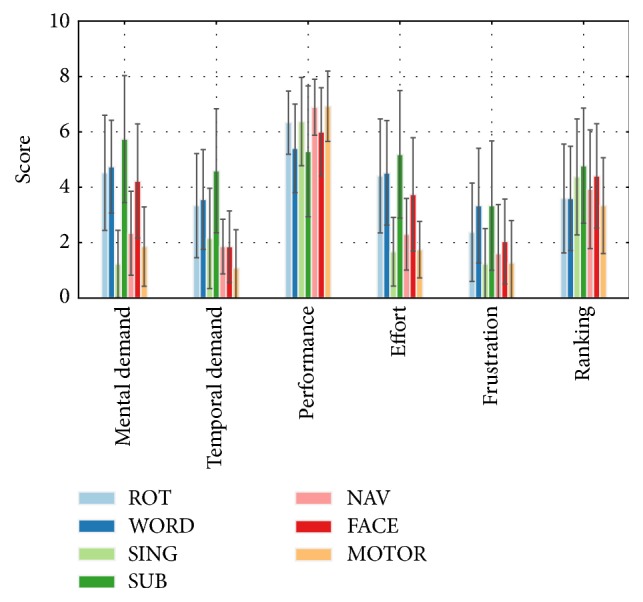
NASA TLX ratings and mental task ranking. Ratings and rankings are averaged over each subject and session. Error bars correspond to the standard deviation of each group of rating and mental task.

The average ratings, as well as the task rankings, are shown in Figure 11. Overall, the SUB task induced the most mental and temporal load, effort, and frustration, led to the poorest perceived performance, and was the least preferred. Other brain teasers (WORD and ROT) induced a medium load, effort, and frustration but were ranked high against other tasks in terms of preference. On the other hand, dynamic imagery tasks (SING, NAV, and MI) induced a relative low load, did not require much effort, and were the least frustrating, while leading to high perceived performance but mixed preference rankings. Finally, the only passive imagery task (FACE) required high mental load and effort but low temporal load and led to slightly lower performance and higher frustration levels, while being ranked among the least preferred tasks.

## 4. Discussion

### 4.1. Optimal Mental Task and Combinations

In this work, we studied seven different mental tasks (mental rotation, word generation, mental subtraction, mental singing, mental navigation, motor imagery, and face imagery) from an electrophysiological and neurohemodynamic perspective in order to uncover the most promising contenders. In the following sections, we discuss the results obtained with each mental task individually and highlight its potential usability in an online BCI.

#### 4.1.1. Mental Rotation

Task pairs with mental rotation were always ranked among the best pairs, in all modality configurations. Pairs including ROT and any task other than SUB yielded peak *κ* higher than average. These results confirm the findings of previous studies [21, 33], in which ROT was also among the best tasks. However, in our study ROT was performed in a slightly different way: participants were shown two figures and had to rotate one to evaluate if it was the same as the other one or a mirrored version, instead of simply imagining a single figure rotating. The introduction of a clear goal puts this task in the brain teaser category with WORD and SUB, which were previously shown to yield high performance. However, the average increase in performance brought in by feature fusion was among the lowest of any tasks.

The features selected for the high performing pairs using ROT were consistently more important at the back of the head in EEG and in the left prefrontal region for NIRS. The ERD/ERS analysis supports this observation: high levels of ERD were observed in the occipital regions in all four plotted bands, suggesting that these regions were recruited during mental rotation. In another study [21], similar ERD patterns were found for the low *β* band between 0.5 and 2 seconds after the beginning of the task, with additional ERD in the prefrontal region. A distinct pattern over the two motor cortices can be seen in both *α* and *β*, suggesting motor imagery might have been used by some participants to help mentally rotate the L-shaped figures. The first findings are confirmed by a review looking at 32 fMRI and Positron Emission Tomography (PET) neuroimaging studies that concluded that the posterior occipital cortex was consistently activated during mental rotation [60]. In this review, the author also notes the activation of focused prefrontal cortex regions and more precisely the left inferior frontal cortex for studies that encouraged motor simulation (i.e., when participants were asked to imagine manipulating the objects to be rotated). Although this was not precisely the case here, participants were not given specific instructions as to how they should perform the rotation, and so some might have used this approach, explaining the strong HbO decrease in the prefrontal regions.

Although ranked as third most demanding, frustrating, and effort-inducing task, ROT was often among the three user-preferred tasks. Therefore, even though ROT might induce fatigue more rapidly than other tasks, its high performance in both NIRS and EEG and its good preference ranking make it an excellent candidate for a BCI.

#### 4.1.2. Word Generation

Word generation led to above average classification performance for all three modality configurations and usually ranked third or fourth among tasks. These results are similar to those observed in EEG for WORD pairs in another study [21]. The fusion proved to be particularly useful for WORD. Indeed, four task pairs with WORD yielded significant *κ* increases with EEG-NIRS fusion.

The selected features were predominantly in the left temporal region in EEG and in the left frontotemporal region in NIRS. In terms of ERD/ERS, this effect was mostly noticeable in the low *α* and high *β* bands, in which the left temporal region undergoes desynchronization. The low *β* pattern characterized by desynchronization in the occipital and central left regions is very similar to the one found in Friedrich et al. (2012) [21]. The temporal regions were also highlighted in NIRS, with strong HbO increase over the left temporal lobe and HbR decrease over both sides. Again, these findings are further confirmed by recent fMRI studies focused on anatomical regions supporting different aspects of language [61]. Indeed, Price found that the left middle frontal cortex and the left pars opercularis were consistently activated during word retrieval tasks. These brain areas overlap with the regions identified in our results.

Similarly to ROT, WORD ranked as the second most demanding, frustrating, and effort-inducing task but was still among the three user-preferred tasks. Contrary to ROT though, participants often rated their performance for the WORD task as low. This might be explained by the purely random selection of the first letter from which words had to be generated: some letters are rarely found at the beginning of a word (such as Z, X, or Q), and so participants who were given these letters probably performed worse. The difficulty of the WORD task would need to be adjusted in a future implementation by limiting the selection of some rarer letters. Overall, word generation is a good candidate for a BCI due to its high performance in both NIRS and EEG and its good subjective evaluation.

#### 4.1.3. Mental Subtraction

Mental subtraction ranked second in terms of average classification performance for all three modality configurations. SUB is the task that benefited the most from the feature fusion, with two of the largest average increases in *κ*. These results confirm the findings of [21] in EEG, but not of [33] in NIRS. Indeed, Hwang et al. found mental subtraction to be on par with other imagery tasks (SING, MI) in terms of how often it led to classification accuracy above 70%. However, the authors defined their SUB task as the successive subtraction of two “simple numbers” (suggesting one-digit numbers) from a three-digit number, which is simpler than the task used in our study. Moreover, a mental multiplication task (of two two-digit numbers), which was not used here, led to the best performance in their work. Since some fMRI studies have found the two operations to be similarly encoded in the brain (although some studies found differences) [62], and since the difficulty level of Hwang et al.'s multiplication task might be closer to our subtraction task, we hypothesize that they could have achieved similar performance with harder subtractions.

The selected features revealed consistent importance at electrode positions PO7 and PO8 in EEG and in the prefrontal and right temporoparietal regions in NIRS. This is also seen in the ERD/ERS patterns as a strong desynchronization in low and high *α* as well as low *β* in the occipitoparietal regions and part of the prefrontal regions. FMRI studies identified the involvement of the precuneus, located in the midline portion of the centroparietal region, and of frontal regions as commonly activated during the execution of different arithmetic operations [63]. Interestingly, in the same study, Fehr et al. found a statistically significant increase in activity in the bilateral inferior parietal regions when comparing a complex subtraction task to a simple one, which could explain these clear patterns of importance for PO7 and PO8 features. As for the prefrontal patterns found in NIRS, they might be related to an increased working memory load, which would result in the activation of the dorsolateral prefrontal cortex [64]. The patterns of HbR decrease in both temporal lobes being very similar to the ones observed in WORD; we hypothesize that participants might have used a speech-based strategy to perform the subtractions.

As a brain teaser, SUB ranked as the most demanding, frustrating, and effort-inducing task, making it the worst perceived for performance and the least preferred task. Before using SUB in a BCI, it might be useful therefore to further assess its optimal difficulty level to avoid tiring or frustrating the user. Additionally, alternative arithmetic operations such as mental multiplication might yield better results and should therefore be studied [33], again at optimal difficulty levels. In either case, SUB can definitely benefit from a EEG-NIRS fusion approach and is thus recommendable if such a system is already in use.

#### 4.1.4. Mental Singing

Task pairs using mental singing led to below average performance for all three modality configurations. Analogous results were obtained for EEG [21] and for NIRS [33] in previous studies comparing many mental tasks. Similarly, in a prefrontal NIRS study, Power et al. (2011) compared the performances of rest-SING and rest-SUB pairs and found that mental singing led to poorer performance on average [28].

It is difficult to determine if consistent brain regions were recruited in the mental singing task based on the feature importance analysis, as in both EEG and NIRS the topographical maps show varying patterns for pairs using SING. Indeed, in EEG, the following regions all showed strong importance: parietooccipital (with ROT), bilateral parietal (with SUB), left parietal and right occipital (with NAV), left temporal (with SING), left motor cortex (with MI), and occipital (with FACE). Similar disparate patterns were found in NIRS. This high variability in importance of brain regions is most likely due to the low discriminability of the physiological processes behind mental singing, especially in the studied modalities, that are essentially limited to cortical structures. Indeed, if little information from singing could be decoded in EEG and NIRS, a classifier would focus on features provided by the second task to make its decision and thus would not choose consistent features across pairs containing SING.

Neuroimaging studies of covert singing identified activation in the frontoparietal regions (bilateral motor cortices and Broca's area) [65, 66]. This could explain the ERD patterns in the low and high *β* band located in the left hemisphere and the similar HbR patterns over the temporal and central lobes. These studies also provide insights into why mental singing did not produce distinguishable patterns: first, Gunji et al. (2007) used MEG to compare the oscillatory processes of humming, speaking, and overt and covert singing and found that covert singing consistently recruited the least cerebral area [66]. A very small area of activation will make it harder for modalities such as EEG and NIRS to pick up relevant activity when restricted to a small number of sensors. Second, in their study of opera singers, Kleber et al. (2007) found significant activation of emotion-related deep brain structures such as the insula, the amygdala, and the hippocampus [65]. Since in our study we specifically asked the participants to focus on the emotions produced by singing a song they personally chose, it is possible that these regions were activated but that the depth limitations of EEG and NIRS prevented these patterns from being measured. The strong *α* and *β* ERS visible in Figure 3 further supports this hypothesis. Indeed, the high increase in these oscillatory processes indicates that most cortical structures were not activated during singing. The strong ERS patterns in the occipital region thus suggest the cortical idling of visual processing centers during mental singing.

SING induced the lowest mental demand, effort, and frustration and was reasonably well ranked by participants. Since pairing SING with brain teasers led to high classification performance, it seems reasonable to use it in a BCI context. Nevertheless, because of the apparent low discriminability of mental singing in EEG and NIRS, it would be important to first assess how it compares to baseline data: if it is not different enough from the baseline, this task could eventually provoke false negatives in a BCI using a no-control state. It is to be noted that this analysis was not performed here due to baseline periods being used for normalization of the features, thus limiting the significance of such an analysis on our dataset.

#### 4.1.5. Mental Navigation

Task pairs using mental navigation led to below average performance for all three modality configurations, making them very similar in terms of performance to pairs including mental singing, and yielded the lowest average *κ* increase in fusion. Similar results were obtained in another study [21] for EEG-only classification, whereas no previous NIRS results were found in the literature.

ERD/ERS patterns for mental navigation showed mostly left hemispheric activation, particularly in the low *α* and low *β* bands. These results are consistent with the low *β* patterns found in Friedrich et al. (2012) above the left hemisphere [21] and are further supported by the results of an fMRI study looking at 16 subjects engaged in mental navigation of familiar places [67]. In their work, Ino et al. reported statistically significant activation in the left premotor area, the left angular gyrus (parietal lobe), and deeper brain structures such as the bilateral retrosplenial areas, parts of the hippocampus, and the cerebellum [67]. These patterns are not easily discerned in the feature importance topographical maps, where different brain regions seem informative for each pair in both EEG and NIRS, as was the case for SING. However, Ino et al. used a more complex navigation task in which participants were instructed to imagine walking through Kyoto while counting the number of turns they made. Since both our study and [21] obtained poor performance with this task, we hypothesize that increasing the difficulty level might have helped produce stronger and clearer activation.

NAV ranked as the third least demanding, frustrating, and effort-inducing task and led to high perceived performance. Ranked as fourth favorite task on average, NAV, like SING, should be considered for implementation in a BCI mostly because it led to good performance when paired with brain teasers. Increasing the difficulty level of the task might however lead to superior performance. Finally, its low *κ* increase in the multimodal case makes it less useful to use with a NIRS-EEG approach.

#### 4.1.6. Motor Imagery

Motor imagery is the most often used mental task in the hBCI literature [11], providing a wealth of previous articles to compare our results to. Whereas MI was the second worst task in NIRS, it achieved third and fourth best performance in EEG-only and EEG-NIRS configurations, respectively. MI was also the second worst task in terms of performance improvement when feature fusion was used. Although using a different processing and classification pipeline, Fazli et al. worked toward a similar hybrid EEG-NIRS-BCI as in our study but only evaluated the use of a left-hand versus right-hand MI paradigm [40]. The authors found that EEG-NIRS fusion could improve the classification accuracy by 5% on average in 90% of subjects, which is hard to compare to our study because of methodological differences. However, we also noted a marked improvement across participants (Δ*κ* = 0.13), even though this improvement was among the lowest in our seven tasks.

Although patterns induced by MI are most likely obscured by those of brain teasers in most pairs (as was the case for SING and NAV), feature importance analysis shows the importance of left and right motor cortex features in NAV-MI, SING-MI, and FACE-MI in EEG. This phenomenon seems to occur only for the NAV-MI pair in NIRS, but this might reveal in fact a pattern proper to NAV, as the NAV-FACE map shows similar feature importance. The ERD/ERS patterns in the first three bands showed activation of the motor cortices, with the left hemisphere being predominant, as expected from the right-hand movement to be imagined. Classical BCI experiments have shown this pattern multiple times [68, 69], and the particular pattern in low *β* is very similar to the one reported in [21]. This is further supported by strong HbO and HbT increases over the left hemisphere. However, desynchronization of both the left and right frontal cortices is visible in the high *β* band, a phenomenon not reported in the aforementioned literature. Similar bilateral frontal ERD patterns were found for almost every task in high *β*, suggesting that there might have been an unexpected brain activity-inducing event affecting this band. As is the case for SING, strong ERS in the low and high *α* bands, predominantly in the occipital lobe, suggests that visual functions were not recruited during motor imagery.

MI induced either the lowest or second lowest demand, effort, and frustration, yielded the highest perceived performance, and was ranked as the most pleasant task on average. Pairing MI with brain teasers led to high classification performance in our case and was shown in the literature to yield good EEG and NIRS performance when paired with another imagery task (such as another MI task performed with the other hand) [40]. We thus conclude that MI is a useful task that deserves its predominant place in the BCI literature.

#### 4.1.7. Face Imagery

Face imagery was the worst task in all modality configurations. Fusion improved the classification performance of FACE, but not differently from most other tasks. These results are in line with those of Friedrich et al. for EEG [21], whereas no reference for NIRS-based classification of face imagery could be found.

The main brain structure known to be activated specifically in response to face stimuli is the fusiform gyrus, found in the posterior part of the cortex [70]. In addition, in reaction to famous faces, activation of subsets of the inferior occipital gyri, lateral fusiform gyri, superior temporal sulcus, and amygdala was identified in [70]. The fact that the main structure recruited (fusiform gyrus) is farther from the cortical layers accessible with EEG and NIRS could certainly account for the difficulty in classifying FACE tasks. This could also explain the disparate feature importance topographical patterns observed in both EEG and NIRS.

The ERD/ERS pattern observed in the low *β* band was similar to the one reported in [21]: desynchronization of the central and frontal lobes, with synchronization of the temporal lobes. Moreover, *α* bands showed distinctly high power in the occipital lobes, which could be explained by some low mental load-induced drowsiness. This is especially relevant since some of the brain structures mentioned above are located in the parietal and occipital brain regions.

Although inducing low temporal demand, the FACE task was almost on par with brain teasers for mental demand and effort, suggesting participants found it difficult to imagine the face of their closest friend. Falling among the three least preferred tasks, FACE is however not a good candidate for a BCI, as it produced consistently low unimodal and multimodal performance, and was generally not particularly appealing to users.

#### 4.1.8. Best Task Pairs

Based on these results, we conclude that out of the 21 task pairs studied in our study combinations of a brain teaser (ROT, SUB, or WORD) and an imagery task (MI, FACE, NAV, and SING) are most likely to yield good performance. This confirms the previous results of previous studies [21, 33]. More specifically, the ROT-MI combination performed the best in our experiments. In the case where a NIRS-EEG system is available for the implementation of the BCI, our results show that pairs based on WORD and NAV might benefit the most from feature fusion.

### 4.2. Evaluation of Fusion Performance in a Realistic Context

A few methodological points would have to be approached differently in a realistic context application. First, the analysis of the effect of fusion on classification performance was done in two parts. The peak *κ* achieved by fusion was compared to the peak *κ* of EEG alone, leading to small increases on the order of Δ*κ* = 0.02. By looking at the evolution of performance across time (Figures 5, 7, and 9), NIRS was found to provide additional information around 11 seconds into the task. We thus chose to report the performance increase for the one-second window between 11 and 12 seconds after stimulus onset to highlight the added value of a fusion approach. In contrast, many NIRS studies extracted the same feature over windows of many different sizes and combined them as different features of the same mental task instance [28–30, 33, 34]. While this approach can hamper classifier performance by increasing the dimensionality of the input, it also provides more information and thus can lead to better accuracy.

Another critical point highlighted in many of the aforementioned studies is the hemodynamic response delay in NIRS. Indeed, in our study, we found it took around 11 seconds for NIRS features to yield peak performance. This is a long time to wait when trying to operate an active BCI, making a unimodal NIRS-BCI poorly usable in most contexts. In turn, some participants saw their EEG performance decrease steadily across a trial, probably due to them being bored or tired, reducing the quality of their mental imagery and solving skills. However, by combining NIRS with EEG, we could reach satisfactory performance in the first seconds after stimulus onset, while still benefiting from increased performance later in the trial.

Despite these limitations, several applications could benefit from the peculiarities of our hBCI paradigm. For example, one could design a BCI where a decision has to be sustained over a few seconds before an output is given to ensure a certain level of certainty. Our system would be useful to recognize a specific mental task over longer periods of time, while also providing increased classification performance.

Another example of how the fusion of EEG and NIRS features might be useful was shown in our analysis for participant S04. Indeed, this participant had the lowest EEG-only performance and did not reach peak *κ* above 0.7 for a single task pair when using EEG features only. However, this participant's NIRS-only performance proved much better, and when EEG and NIRS were combined S04 benefited from the largest performance increase across participants and achieved high performance in seven task pairs. This pattern was observed for many participants: usually, one modality led to better performance than the other, and combining both EEG and NIRS allowed improvements in many task pairs. For example, this means a BCI user with poor EEG-only control would still be able to achieve high performance, thus potentially helping tackle the so-called BCI illiteracy problem.

## 5. Conclusion

In this work, we investigated the use of two noninvasive functional neuroimaging techniques, EEG and NIRS, for the binary classification of seven different mental tasks. We identified optimal mental task pairs across nine participants, for EEG-only, NIRS-only, and EEG-NIRS fusion classification schemes, and assessed the impact of a multimodal approach on the classification performance.

Pairs formed of a brain teaser, that is, a mental task that requires problem-solving skills (ROT, SUB, and WORD), and an imagery task (MI, FACE, and SING) consistently yielded the best classification performance for unimodal EEG and NIRS schemes, as well as for a multimodal fusion scheme. In contrast to unimodal performance results on par with those of previous reports, the multimodal approach led to an average increase of 0.03 in peak Cohen's *κ* when using features extracted from one-second windows (equivalent to a 1.5% accuracy increase in balanced settings). Similarly, a 0.20 increase in *κ* (10% accuracy increase) was obtained when focusing on the optimal NIRS windows.

The analysis of the trained classification models unveiled interesting spatial patterns of brain activity and the importance of feature subtypes in modality fusion. Particularly, *α*- and *β*-related EEG features proved to be the most useful, followed by HbO or HbR amplitude NIRS features, depending on the individual. The occipital and parietal regions yielded the most important EEG features, whereas NIRS features extracted from the prefrontal and frontal regions were the most informative. Our proposed feature analysis approach made it possible to delve deeper into the classification results, and shed light on the role of different neurophysiological modalities toward more efficient and flexible BCIs.

An important future research direction should be the implementation of online BCIs based on the optimal mental task pairs we identified. The restrictions induced by a real-time implementation (setup time, computing efficacy, robustness to noise, etc.) will all have to be overcome to yield a truly usable brain-computer interface.

## Figures and Tables

**Table 1:** Questionnaire items and description based on the NASA TLX test. The first five questions were given a rating between 1 and 10.

Dimension	Question
Mental demand	How mentally demanding was the task?
Temporal demand	How hurried or rushed was the pace of the task?
Performance	How successful were you in accomplishing what you were asked to do?
Effort	How hard did you have to work to accomplish your level of performance?
Frustration	How insecure, discouraged, irritated, stressed and annoyed were you?
Task ranking	Which are your preferred tasks, in order of importance?

**Table 2:** EEG-only peak *κ* for each subject and task pair, when using features extracted from one-second windows. Task pairs and participants are shown in descending order of average *κ*, from top to bottom and left to right, respectively. *κ* values between 0.4 and 0.7 (satisfactory performance) are highlighted in italic font, whereas kappa values greater than 0.7 (high performance) are  highlighted in bold font. The average peak time with standard deviation is also included for each task pair. ^*∗*^*p* < 0.05, ^*∗∗*^*p* < 0.01, ^*∗∗∗*^*p* < 0.001.

	S10	S03	S07	S06	S05	S11	S01	S09	S04	Average	Peak time (s)
ROT-MI	** 0.93 **	** 0.89 **	** 0.87 **	** 0.95 **	** 0.84 **	** 0.80 **	** 0.77 **	** 0.77 **	* 0.61 *	** 0.83 **±** 0.10**^**∗∗∗**^	3.83 ± 3.3
ROT-FACE	** 0.86 **	** 0.86 **	** 0.84 **	** 0.94 **	** 0.92 **	* 0.68 *	** 0.79 **	* 0.66 *	* 0.51 *	** 0.79 **±** 0.14**^**∗∗∗**^	2.61 ± 1.0
ROT-SING	** 1.00 **	** 0.80 **	** 0.86 **	** 0.81 **	** 0.90 **	** 0.71 **	** 0.81 **	* 0.56 *	* 0.53 *	** 0.78 **±** 0.15**^**∗∗**^	5.61 ± 4.4
ROT-NAV	** 0.96 **	** 0.72 **	** 0.83 **	* 0.61 *	* 0.68 *	* 0.58 *	** 0.75 **	* 0.58 *	* 0.63 *	* 0.70 *±* 0.13*^**∗∗**^	4.06 ± 1.0
SUB-MI	** 0.91 **	** 0.89 **	** 0.82 **	** 0.91 **	** 0.81 **	** 0.74 **	** 0.70 **	** 0.85 **	0.39	** 0.78 **±** 0.16**^**∗∗**^	4.28 ± 2.1
SING-SUB	** 0.99 **	** 0.72 **	** 0.73 **	** 0.82 **	** 0.89 **	* 0.65 *	** 0.77 **	* 0.68 *	*0.42 *	** 0.74 **±** 0.16**^**∗∗**^	2.50 ± 1.6
SUB-FACE	* 0.65 *	** 0.84 **	** 0.71 **	** 0.88 **	** 0.87 **	* 0.62 *	** 0.76 **	* 0.68 *	0.37	** 0.71 **±** 0.16**^**∗∗**^	6.39 ± 5.0

ROT-WORD	** 0.96 **	** 0.74 **	* 0.69 *	* 0.61 *	** 0.90 **	* 0.62 *	* 0.56 *	* 0.50 *	* 0.53 *	* 0.68 *±* 0.16*^*∗*^	5.94 ± 5.3
NAV-MI	** 0.78 **	** 0.86 **	** 0.78 **	** 0.81 **	* 0.66 *	* 0.66 *	* 0.60 *	* 0.46 *	* 0.41 *	* 0.67 *±* 0.16*^*∗*^	5.61 ± 5.3
WORD-MI	** 0.96 **	* 0.61 *	** 0.75 **	** 0.88 **	* 0.57 *	* 0.64 *	* 0.58 *	* 0.63 *	* 0.44 *	* 0.67 *±* 0.16*^*∗*^	5.83 ± 3.8
WORD-FACE	** 0.75 **	** 0.73 **	*0.67 *	** 0.87 **	** 0.73 **	* 0.64 *	* 0.65 *	* 0.44 *	0.36	* 0.65 *±* 0.16*^*∗*^	6.06 ± 3.9
SING-NAV	** 0.83 **	* 0.69 *	** 0.72 **	* 0.69 *	** 0.72 **	* 0.64 *	** 0.74 **	0.27	* 0.43 *	* 0.64 *±* 0.17*^*∗*^	3.39 ± 2.1
SUB-NAV	** 0.88 **	* 0.59 *	** 0.71 **	* 0.46 *	* 0.69 *	* 0.46 *	* 0.55 *	* 0.66 *	0.33	* 0.59 *±* 0.16 *	3.61 ± 5.8
WORD-SING	** 0.88 **	* 0.66 *	** 0.73 **	** 0.84 **	* 0.53 *	* 0.53 *	** 0.73 **	0.36	0.35	* 0.62 *±* 0.19 *	5.61 ± 6.6

ROT-SUB	** 0.72 **	* 0.60 *	* 0.66 *	0.39	0.38	* 0.53 *	* 0.66 *	* 0.50 *	* 0.43 *	* 0.54 *±* 0.13 *	4.06 ± 5.2
WORD-SUB	** 0.88 **	* 0.69 *	* 0.42 *	* 0.63 *	** 0.75 **	0.37	* 0.40 *	* 0.68 *	*0.44 *	* 0.58 *±* 0.18 *	3.94 ± 2.9
NAV-FACE	* 0.53 *	** 0.79 **	* 0.54 *	** 0.80 **	** 0.82 **	* 0.49 *	* 0.44 *	0.28	0.28	* 0.55 *±* 0.21 *	5.39 ± 4.8
WORD-NAV	** 0.79 **	* 0.55 *	* 0.63 *	* 0.41 *	* 0.46 *	* 0.50 *	* 0.48 *	0.35	0.30	* 0.50 *±* 0.15 *	3.17 ± 2.8
SING-MI	** 0.78 **	* 0.56 *	0.39	* 0.47 *	0.36	* 0.41 *	0.25	0.38	0.40	* 0.44 *±* 0.15 *	2.39 ± 2.4
FACE-MI	* 0.62 *	* 0.60 *	0.37	* 0.42 *	0.35	* 0.49 *	0.21	0.35	*0.51 *	* 0.44 *±* 0.13 *	1.61 ± 2.2
SING-FACE	* 0.63 *	* 0.47 *	0.30	* 0.50 *	0.39	0.28	0.25	0.27	0.22	0.37 ± 0.14	5.06 ± 5.5
Average	** 0.82 **	** 0.71 **	* 0.67 *	** 0.70 **	* 0.68 *	* 0.57 *	* 0.59 *	* 0.52 *	* 0.42 *	* 0.63 *±* 0.16 *	4.33 ± 3.7

**Table:** (a) EEG only

	ROT-MI	ROT-FACE	ROT-SING	SUB-MI	SING-SUB
(1)	pwr_high_alpha_O2	pwr_high_alpha_O2	pwr_t4_30_P8	pwr_high_alpha_PO7	pwr_alpha/beta_PO8
(2)	pwr_low_alpha_CP6	pwr_t4_30_P10	pwr_t4_30_O2	pwr_alpha/beta_Iz	pwr_total_PO7
(3)	pwr_high_alpha_CP6	pwr_alpha/beta_O1	pwr_high_alpha_PO8	pwr_total_PO8	pwr_high_alpha_Iz
(4)	pwr_high_alpha_O1	pwr_high_alpha_PO7	pwr_t4_30_P10	pwr_theta/beta_O2	pwr_total_PO8
(5)	pwr_t4_30_P8	pwr_low_beta_P7	pwr_alpha/beta_PO8	pwr_theta/beta_P9	pwr_total_P7

**Table:** (b) NIRS only

	ROT-MI	ROT-SING	ROT-FACE	SUB-MI	ROT-WORD
(1)	mean_S15-D1_HbO	mean_S15-D1_HbO	mean_S15-D1_HbO	mean_S14-D22_HbR	mean_S15-D5_HbO
(2)	mean_S10-D17_HbR	mean_S1-D2_HbT	mean_S10-D14_HbO	mean_S15-D1_HbR	mean_S13-D24_HbR
(3)	mean_S1-D2_HbT	mean_S6-D9_HbR	mean_S3-D6_HbR	mean_S15-D5_HbT	mean_S16-D17_HbR
(4)	mean_S13-D24_HbR	mean_S13-D22_HbR	mean_S10-D17_HbO	mean_S15-D1_HbO	mean_S13-D22_HbO
(5)	mean_S14-D24_HbR	mean_S14-D24_HbR	mean_S10-D17_HbR	mean_S1-D1_HbO	mean_S13-D24_HbO

**Table:** (c) EEG-NIRS fusion

	ROT-FACE	ROT-MI	ROT-SING	SUB-MI	SING-SUB
(1)	mean_S15-D1_HbO	pwr_theta/beta_O2	mean_S15-D1_HbO	mean_S14-D22_HbR	pwr_high_alpha_O2
(2)	pwr_t4_30_O2	pwr_high_alpha_O1	pwr_high_alpha_O2	pwr_high_alpha_PO7	pwr_t4_30_P9
(3)	pwr_theta/beta_O2	pwr_t4_30_O2	pwr_t4_30_P9	pwr_t4_30_O2	pwr_high_alpha_P10
(4)	pwr_theta/beta_PO7	pwr_t4_30_PO8	pwr_t4_30_O2	pwr_high_alpha_O2	mean_S15-D1_HbO
(5)	pwr_high_alpha_PO8	mean_S14-D24_HbR	pwr_theta/beta_O1	pwr_high_alpha_PO8	pwr_low_alpha_P9

**Table 4:** NIRS-only peak *κ* values for each subject and task pair, when using features extracted from one-second windows. Task pairs and participants are shown in descending order of average *κ*, from top to bottom and left to right, respectively. *κ* values between 0.4 and 0.7 (satisfactory performance) are  highlighted in italic font, whereas *κ* values greater than 0.7 (high performance) are shown in bold font. The average peak time with standard deviation is also included for each task pair. ^*∗*^*p* < 0.05

	S10	S07	S04	S11	S09	S06	S01	S05	S03	Average	Peak time (s)
ROT-SING	** 0.80 **	* 0.66 *	** 0.73 **	* 0.61 *	* 0.59 *	* 0.55 *	* 0.64 *	* 0.42 *	* 0.52 *	* 0.61 *±* 0.11*^*∗*^	13.28 ± 3.6
SING-SUB	* 0.69 *	* 0.60 *	* 0.59 *	* 0.60 *	* 0.68 *	* 0.46 *	*0.50 *	* 0.42 *	* 0.53 *	* 0.56 *±* 0.09*^*∗*^	12.39 ± 2.6
ROT-MI	** 0.81 **	** 0.74 **	** 0.72 **	* 0.60 *	* 0.57 *	* 0.68 *	* 0.62 *	0.32	* 0.53 *	* 0.62 *±* 0.14*^*∗*^	12.61 ± 4.7
SUB-MI	** 0.83 **	* 0.60 *	* 0.57 *	** 0.72 **	** 0.77 **	* 0.57 *	* 0.57 *	0.33	* 0.42 *	* 0.60 *±* 0.16 *	12.06 ± 2.5
ROT-NAV	** 0.80 **	** 0.74 **	** 0.73 **	* 0.53 *	* 0.50 *	* 0.68 *	* 0.49 *	0.40	0.38	* 0.58 *±* 0.16 *	12.72 ± 3.0
ROT-WORD	** 0.89 **	* 0.66 *	** 0.76 **	* 0.63 *	* 0.62 *	* 0.55 *	* 0.58 *	* 0.42 *	0.28	* 0.60 *±* 0.18 *	12.72 ± 2.7
SUB-FACE	** 0.78 **	* 0.60 *	** 0.76 **	* 0.66 *	* 0.62 *	* 0.48 *	* 0.53 *	0.33	0.33	* 0.57 *±* 0.16 *	13.39 ± 4.2

ROT-FACE	** 0.80 **	* 0.51 *	** 0.84 **	* 0.53 *	* 0.48 *	* 0.66 *	* 0.70 *	* 0.48 *	0.18	* 0.58 *±* 0.20 *	14.72 ± 3.2
WORD-SUB	* 0.67 *	*0*.*53*	*0.63*	* 0.60 *	*0.53*	0.36	* 0.41 *	*0.43 *	0.24	* 0.49 *±* 0.14 *	14.61 ± 4.9
WORD-SING	** 0.75 **	** 0.76 **	* 0.50 *	* 0.60 *	0.24	0.39	* 0.52 *	0.36	* 0.44 *	* 0.51 *±* 0.18 *	13.94 ± 2.8
SUB-NAV	** 0.87 **	*0.42 *	* 0.45 *	* 0.64 *	* 0.66 *	0.33	* 0.44 *	0.37	0.39	* 0.51 *±* 0.18 *	9.94 ± 3.8
ROT-SUB	* 0.53 *	* 0.43 *	* 0.43 *	* 0.58 *	0.35	* 0.56 *	0.27	* 0.56 *	0.36	* 0.45 *±* 0.11 *	13.39 ± 3.7
WORD-FACE	** 0.78 **	* 0.61 *	* 0.53 *	** 0.72 **	0.12	* 0.41 *	0.25	* 0.66 *	0.29	* 0.48 *±* 0.23 *	10.50 ± 6.0
WORD-MI	** 0.74 **	** 0.72 **	* 0.54 *	* 0.66 *	0.40	0.39	0.28	0.32	0.16	* 0.47 *±* 0.21 *	8.94 ± 6.7

WORD-NAV	**0.84 **	** 0.74 **	* 0.44 *	* 0.53 *	0.34	0.33	0.31	0.28	0.28	* 0.45 *±* 0.21*	7.83 ± 5.8
SING-NAV	0.32	* 0.52 *	0.35	0.28	0.32	0.27	0.32	0.28	* 0.48 *	0.35 ± 0.09	12.94 ± 3.1
NAV-MI	* 0.66 *	0.13	0.31	0.33	0.34	0.28	0.30	0.20	0.29	0.32 ± 0.15	11.83 ± 3.2
SING-MI	0.39	* 0.42 *	0.29	0.33	0.25	0.32	0.30	0.03	* 0.41 *	0.30 ± 0.12	12.50 ± 3.2
SING-FACE	* 0.43 *	* 0.46 *	0.16	0.02	0.20	0.33	0.05	0.09	* 0.41 *	0.24 ± 0.17	6.17 ± 7.4
NAV-FACE	* 0.57 *	0.20	0.36	0.30	0.29	0.21	0.17	0.25	0.17	0.28 ± 0.13	7.39 ± 6.8
FACE-MI	* 0.40 *	0.30	* 0.43 *	0.28	0.19	0.13	0.26	0.10	0.01	0.23 ± 0.14	8.06 ± 5.2
Average	** 0.82 **	* 0.67 *	* 0.42 *	* 0.57 *	* 0.52 *	** 0.70 **	* 0.59 *	* 0.68 *	** 0.71 **	* 0.47 *±* 0.15 *	11.52 ± 4.2

**Table 5:** EEG-NIRS fusion peak *κ* values for each subject and task pair, when using features extracted from one-second windows. Task pairs and participants are shown in descending order of average *κ*, from top to bottom and left to right, respectively. *κ* values between 0.4 and 0.7 (satisfactory performance) are highlighted  in italic font, whereas *κ* values greater than 0.7 (high performance) are shown in bold font. average peak time with standard deviation are also included for each task pair. ^*∗*^*p* < 0.05, ^*∗∗*^*p* < 0.01, ^*∗∗∗*^*p* < 0.001.

	S10	S03	S07	S06	S11	S05	S01	S04	S09	Average	Peak time (s)
ROT-MI	** 0.93 **	** 0.88 **	** 0.88 **	** 0.95 **	** 0.78 **	** 0.83 **	** 0.77 **	** 0.75 **	** 0.77 **	** 0.84 ± 0.08** ^**∗∗∗**^	9.83 ± 5.8
SUB-FACE	** 0.78 **	** 0.83 **	*0.69 *	** 0.87 **	** 0.74 **	** 0.90 **	** 0.77 **	** 0.76 **	* 0.68 *	** 0.78 ± 0.07** ^**∗∗∗**^	4.61 ± 3.5
ROT-FACE	** 0.85 **	** 0.86 **	** 0.85 **	** 0.95 **	* 0.69 *	** 0.92 **	** 0.78 **	** 0.86 **	* 0.69 *	** 0.83 ± 0.09** ^**∗∗∗**^	9.39 ± 5.0
ROT-SING	** 1.00 **	** 0.80 **	** 0.86 **	** 0.81 **	** 0.70 **	** 0.90 **	** 0.81 **	** 0.83 **	* 0.59 *	** 0.81 ± 0.11** ^**∗∗∗**^	6.39 ± 4.4
SUB-MI	** 0.92 **	** 0.90 **	** 0.82 **	** 0.91 **	** 0.78 **	** 0.83 **	** 0.71 **	* 0.55 *	** 0.85 **	** 0.81 ± 0.12** ^**∗∗∗**^	5.17 ± 3.5
SING-SUB	** 0.99 **	** 0.81 **	** 0.75 **	** 0.83 **	* 0.67 *	** 0.88 **	** 0.76 **	* 0.59 *	** 0.74 **	** 0.78 ± 0.12** ^**∗∗∗**^	5.28 ± 4.4
ROT-NAV	** 0.96 **	** 0.73 **	** 0.85 **	* 0.66 *	* 0.60 *	* 0.68 *	** 0.74 **	** 0.72 **	* 0.61 *	** 0.73 ± 0.11** ^**∗∗∗**^	7.72 ± 5.3

ROT-WORD	** 0.96 **	** 0.74 **	** 0.72 **	* 0.65 *	* 0.66 *	** 0.90 **	* 0.61 *	** 0.75 **	* 0.61 *	** 0.73 ± 0.12** ^**∗∗∗**^	9.17 ± 6.9
WORD-FACE	** 0.78 **	** 0.74 **	** 0.71 **	** 0.87 **	** 0.78 **	** 0.76 **	* 0.64 *	*0.51 *	* 0.44 *	* 0.69 *±* 0.14*^*∗∗*^	8.72 ± 5.7
ROT-SUB	** 0.74 **	* 0.58 *	* 0.66 *	* 0.51 *	* 0.56 *	* 0.52 *	* 0.67 *	* 0.51 *	* 0.49 *	* 0.58 *±* 0.09*^*∗∗*^	7.61 ± 3.7
WORD-SUB	** 0.89 **	** 0.71 **	* 0.55 *	* 0.62 *	* 0.60 *	** 0.75 **	* 0.41 *	* 0.69 *	* 0.67 *	* 0.66 *±* 0.13*^*∗∗*^	7.28 ± 5.3
WORD-MI	** 0.96 **	* 0.60 *	** 0.81 **	** 0.88 **	** 0.77 **	* 0.53 *	* 0.56 *	* 0.53 *	* 0.63 *	* 0.70 *±* 0.16*^*∗∗*^	6.94 ± 4.7
NAV-MI	** 0.82 **	** 0.86 **	** 0.75 **	** 0.81 **	* 0.66 *	* 0.68 *	* 0.61 *	* 0.43 *	* 0.45 *	* 0.67 *±* 0.15*^*∗∗*^	1.50 ± 4.5
SUB-NAV	** 0.89 **	* 0.59 *	** 0.73 **	* 0.46 *	* 0.66 *	** 0.70 **	* 0.58 *	* 0.42 *	** 0.72 **	* 0.64 *±* 0.14*^*∗∗*^	6.06 ± 7.1

WORD-SING	** 0.88 **	* 0.66 *	** 0.80 **	** 0.83 **	* 0.60 *	* 0.52 *	** 0.73 **	* 0.57 *	0.31	* 0.66 *±* 0.18*^*∗*^	5.94 ± 4.0
SING-NAV	** 0.80 **	* 0.68 *	** 0.73 **	* 0.68 *	* 0.62 *	** 0.74 **	** 0.71 **	* 0.41 *	0.27	* 0.63 *±* 0.17*^*∗*^	5.06 ± 4.0
WORD-NAV	** 0.80 **	* 0.56 *	* 0.68 *	* 0.42 *	* 0.53 *	* 0.48 *	* 0.48 *	* 0.49 *	0.37	* 0.53 *±* 0.13 *	6.39 ± 4.5
NAV-FACE	* 0.54 *	** 0.77 **	* 0.55 *	** 0.79 **	* 0.49 *	** 0.82 **	* 0.42 *	0.35	0.21	* 0.55 *±* 0.21 *	4.50 ± 4.5
SING-MI	** 0.78 **	* 0.54 *	* 0.41 *	* 0.55 *	0.39	0.36	0.24	*0.43 *	0.39	* 0.46 *±* 0.15 *	4.39 ± 4.8
FACE-MI	* 0.61 *	* 0.56 *	0.37	0.39	* 0.46 *	0.30	0.18	*0*.*51*	0.38	* 0.42 *±* 0.13 *	2.17 ± 2.7
SING-FACE	* 0.63 *	* 0.46 *	* 0.43 *	* 0.44 *	0.25	0.32	0.31	0.21	0.26	0.37 ± 0.13	2.39 ± 2.5
Average	** 0.83 **	** 0.71 **	* 0.70 *	** 0.71 **	* 0.62 *	* 0.68 *	* 0.60 *	* 0.56 *	* 0.53 *	* 0.66 *±* 0.13 *	6.02 ± 4.6

**Table 6:** Change in peak *κ* when adding NIRS features to EEG features, for the models trained on the windows that yielded the highest NIRS *κ*. Task pairs and participants are shown in descending order of average Δ*κ*, from top to bottom and left to right, respectively. Δ*κ* values between 0.4 and 0.7 are  highlighted in italic font, values greater than 0.7 are shown in bold font, and values below 0 are shown in underline font.  ^*∗*^*p* < 0.05.

	S04	S11	S07	S09	S01	S10	S05	S03	S06	Average
SUB-NAV	0.36	0.35	0.02	0.32	0.07	0.33	0.35	0.10	0.11	0.22 ± 0.14^*∗*^
ROT-WORD	* 0.40 *	* 0.56 *	* 0.42 *	* 0.41 *	* 0.48 *	−0.00	0.14	0.12	0.16	0.30 ± 0.20^*∗*^
NAV-FACE	* 0.40 *	0.38	0.37	0.29	0.20	0.20	−0.00	0.23	0.00	0.23 ± 0.15^*∗*^
ROT-NAV	* 0.53 *	0.16	0.21	0.28	0.20	0.09	0.27	−0.01	0.22	0.22 ± 0.15^*∗*^
WORD-NAV	* 0.42 *	0.30	0.34	0.01	0.26	0.16	0.07	0.02	* 0.45 *	0.23 ± 0.17
WORD-SING	0.22	0.20	0.34	0.37	* 0.64 *	* 0.64 *	0.03	0.12	0.02	0.29 ± 0.23
WORD-SUB	** 0.79 **	* 0.58 *	0.13	0.27	0.11	0.24	0.32	−0.06	* 0.45 *	0.31 ± 0.26

ROT-SING	* 0.46 *	0.31	0.06	0.25	0.20	0.18	0.11	0.00	0.03	0.18 ± 0.15
SING-SUB	* 0.55 *	0.12	*0.63 *	0.27	−0.01	** 0.84 **	*0.47 *	0.07	−0.02	0.32 ± 0.31
NAV-MI	0.02	0.14	0.11	0.04	0.02	0.08	0.02	0.19	−0.00	0.07 ± 0.07
SING-NAV	0.17	0.14	*0.59 *	0.20	*0.46 *	0.16	0.01	0.08	−0.00	0.20 ± 0.20
ROT-SUB	0.18	* 0.46 *	−0.05	0.02	0.15	−0.01	*0.61 *	0.21	* 0.47 *	0.23 ± 0.24
SING-MI	0.15	0.36	0.39	0.02	0.22	−0.02	0.09	*0.53 *	−0.03	0.19 ± 0.20
ROT-MI	0.24	0.38	0.05	0.16	0.09	0.00	−0.01	0.01	0.22	0.13 ± 0.13

WORD-FACE	* 0.49 *	* 0.67 *	0.23	−0.07	0.05	0.32	0.18	0.13	−0.00	0.22 ± 0.24
SUB-FACE	** 0.74 **	0.16	* 0.63 *	0.09	0.17	0.11	0.04	0.13	0.00	0.23 ± 0.27
SING-FACE	0.17	−0.04	0.24	0.27	0.03	0.40	0.07	0.02	−0.03	0.13 ± 0.15
ROT-FACE	* 0.62 *	0.30	0.04	0.10	0.21	0.18	0.04	−0.02	−0.01	0.16 ± 0.20
SUB-MI	* 0.67 *	0.33	* 0.62 *	0.23	0.14	0.05	0.02	−0.11	−0.01	0.22 ± 0.28
WORD-MI	* 0.57 *	0.32	0.24	−0.07	0.04	0.00	0.03	0.03	−0.03	0.12 ± 0.21
FACE-MI	0.15	0.03	0.34	−0.00	0.15	0.01	0.03	−0.10	−0.00	0.07 ± 0.13
Average	0.40	0.30	0.28	0.17	0.18	0.19	0.14	0.08	0.09	0.20 ± 0.19

## References

[1] Wolpaw J. R., Birbaumer N., McFarland D. J., Pfurtscheller G., Vaughan T. M. (2002). Brain-computer interfaces for communication and control. *Clinical Neurophysiology*.

[2] Wolpaw J. R., Wolpaw E. W. (2012). Brain-computer interfaces: something new under the sun. *Brain-Computer Interfaces: Principles and Practice*.

[3] Van Erp J. B. F., Lotte F., Tangermann M. (2012). Brain-computer interfaces: beyond medical applications. *Computer*.

[4] Coyle S. M., Ward T. E., Markham C. M., McDarby G. (2004). On the suitability of near-infrared (NIR) systems for next-generation brain-computer interfaces. *Physiological Measurement*.

[5] Hwang H.-J., Kim S., Choi S., Im C.-H. (2013). EEG-based brain-computer interfaces: a thorough literature survey. *International Journal of Human-Computer Interaction*.

[6] Leuthardt E. C., Schalk G., Wolpaw J. R., Ojemann J. G., Moran D. W. (2004). A brain-computer interface using electrocorticographic signals in humans. *Journal of Neural Engineering*.

[7] Mellinger J., Schalk G., Braun C. (2007). An MEG-based brain-computer interface (BCI). *NeuroImage*.

[8] Sitaram R., Caria A., Birbaumer N. (2009). Hemodynamic brain-computer interfaces for communication and rehabilitation. *Neural Networks*.

[9] Sitaram R., Caria A., Veit R. (2007). FMRI brain-computer interface: A tool for neuroscientific research and treatment. *Computational Intelligence and Neuroscience*.

[10] Naseer N., Hong K. (2015). fNIRS-based brain-computer interfaces: a review. *Frontiers in Human Neuroscience*.

[11] Banville H., Falk T. (2016). Recent advances and open challenges in hybrid brain-computer interfacing: a technological review of non-invasive human research. *Brain-Computer Interfaces*.

[12] Pfurtscheller G., Allison B. Z., Bauernfeind G. (2010). The hybrid BCI. *Frontiers in Neuroscience*.

[13] Müller-Putz G. R., Leeb R., Millán J. d. (2013). Principles of hybrid brain–computer interfaces. *Towards Practical Brain-Computer Interfaces*.

[14] Cao T., Wan F., Wong C. M., da Cruz J. N., Hu Y. (2014). Objective evaluation of fatigue by EEG spectral analysis in steady-state visual evoked potential-based brain-computer interfaces. *BioMedical Engineering Online*.

[15] Obermaier B., Neuper C., Guger C., Pfurtscheller G. (2001). Information transfer rate in a five-classes brain-computer interface. *IEEE Transactions on Neural Systems and Rehabilitation Engineering*.

[16] Curran E., Sykacek P., Stokes M. (2004). Cognitive Tasks for Driving a Brain-Computer Interfacing System: A Pilot Study. *IEEE Transactions on Neural Systems and Rehabilitation Engineering*.

[17] Sepulveda F., Dyson M., Gan J. Q., Tsui C. L. A comparison of mental task combinations for asynchronous EEG-based BCIs.

[18] Faradji F., Ward R. K., Birch G. E. A brain-computer interface based on mental tasks with a zero false activation rate.

[19] Dobrea M.-C., Dobrea D. M. The selection of proper discriminative cognitive tasks - A necessary prerequisite in high-quality BCI applications.

[20] Friedrich E. V. C., Scherer R., Sonnleitner K., Neuper C. (2011). Impact of auditory distraction on user performance in a brain-computer interface driven by different mental tasks. *Clinical Neurophysiology*.

[21] Friedrich E. V. C., Scherer R., Neuper C. (2012). The effect of distinct mental strategies on classification performance for brain-computer interfaces. *International Journal of Psychophysiology*.

[22] Scherer R., Faller J., Balderas D. (2013). Brain-computer interfacing: more than the sum of its parts. *Soft Computing*.

[23] Friedrich E. V. C., Scherer R., Neuper C. (2013). Long-term evaluation of a 4-class imagery-based brain-computer interface. *Clinical Neurophysiology*.

[24] Friedrich E. V. C., Scherer R., Neuper C. (2013). Stability of event-related (de-) synchronization during brain-computer interface-relevant mental tasks. *Clinical Neurophysiology*.

[25] Friedrich E. V. C., Neuper C., Scherer R. (2013). Whatever works: a systematic user-centered training protocol to optimize brain-computer interfacing individually. *PLoS ONE*.

[26] Hoshi Y., Tamura M. (1997). Near-infrared optical detection of sequential brain activation in the prefrontal cortex during mental tasks. *NeuroImage*.

[27] Abibullaev B., An J., Moon J.-I. (2011). Neural network classification of brain hemodynamic responses from four mental tasks. *International Journal of Optomechatronics*.

[28] Power S. D., Kushki A., Chau T. (2011). Towards a system-paced near-infrared spectroscopy brain-computer interface: differentiating prefrontal activity due to mental arithmetic and mental singing from the no-control state. *Journal of Neural Engineering*.

[29] Falk T. H., Guirgis M., Power S., Chau T. T. (2011). Taking NIRS-BCIs outside the lab: towards achieving robustness against environment noise. *IEEE Transactions on Neural Systems and Rehabilitation Engineering*.

[30] Power S. D., Kushki A., Chau T. (2012). Intersession consistency of single-trial classification of the prefrontal response to mental arithmetic and the no-control state by NIRS. *PLoS ONE*.

[31] Herff C., Heger D., Putze F., Hennrich J., Fortmann O., Schultz T. Classification of mental tasks in the prefrontal cortex using fNIRS.

[32] Schudlo L. C., Power S. D., Chau T. (2013). Dynamic topographical pattern classification of multichannel prefrontal NIRS signals. *Journal of Neural Engineering*.

[33] Hwang H.-J., Lim J.-H., Kim D.-W., Im C.-H. (2014). Evaluation of various mental task combinations for near-infrared spectroscopy-based brain-computer interfaces. *Journal of Biomedical Optics*.

[34] Schudlo L. C., Chau T. (2015). Single-trial classification of near-infrared spectroscopy signals arising from multiple cortical regions. *Behavioural Brain Research*.

[35] Myrden A., Kushki A., Sejdić E., Chau T. (2012). Towards increased data transmission rate for a three-class metabolic brain-computer interface based on transcranial Doppler ultrasound. *Neuroscience Letters*.

[36] Aleem I., Chau T. (2013). Towards a hemodynamic BCI using transcranial Doppler without user-specific training data. *Journal of Neural Engineering*.

[37] Faress A., Chau T. (2013). Towards a multimodal brain-computer interface: combining fNIRS and fTCD measurements to enable higher classification accuracy. *NeuroImage*.

[38] Nawa N. E., Ando H. (2014). Classification of self-driven mental tasks from whole-brain activity patterns. *PLoS ONE*.

[39] Hortal E., Planelles D., Costa A. (2015). SVM-based brain-machine interface for controlling a robot arm through four mental tasks. *Neurocomputing*.

[40] Fazli S., Mehnert J., Steinbrink J. (2012). Enhanced performance by a hybrid NIRS-EEG brain computer interface. *NeuroImage*.

[41] Khan M. J., Hong M. J., Hong K.-S. (2014). Decoding of four movement directions using hybrid NIRS-EEG brain-computer interface. *Frontiers in Human Neuroscience*.

[42] Koo B., Lee H.-G., Nam Y. (2015). A hybrid NIRS-EEG system for self-paced brain computer interface with online motor imagery. *Journal of Neuroscience Methods*.

[43] Zimmermann R., Marchal-Crespo L., Edelmann J. (2013). Detection of motor execution using a hybrid fNIRS-biosignal BCI: a feasibility study. *Journal of NeuroEngineering and Rehabilitation*.

[44] Banville H. (2015). *HyBrid Brain-Computer Interfaces: Improving Mental Task Classification Performance through Fusion of Neurophysiological Modalities [Master, thesis]*.

[45] Hart S. G., Staveland L. E. (1988). Development of NASA-TLX (Task Load Index): results of empirical and theoretical research. *Advances in Psychology*.

[46] Cegarra J., Morgado N. Étude des propriétés de la version francophone du NASATLX.

[47] Delorme A., Makeig S. (2004). EEGLAB: an open source toolbox for analysis of single-trial EEG dynamics including independent component analysis. *Journal of Neuroscience Methods*.

[48] Bell A. J., Sejnowski T. J. (1995). An information-maximization approach to blind separation and blind deconvolution. *Neural Computation*.

[49] Mognon A., Jovicich J., Bruzzone L., Buiatti M. (2011). ADJUST: an automatic EEG artifact detector based on the joint use of spatial and temporal features. *Psychophysiology*.

[50] Pfurtscheller G., da Silva F. H. L. (1999). Event-related EEG/MEG synchronization and desynchronization: basic principles. *Clinical Neurophysiology*.

[51] Kalcher J., Pfurtscheller G. (1995). Discrimination between phase-locked and non-phase-locked event-related EEG activity. *Electroencephalography and Clinical Neurophysiology*.

[52] Pfurtscheller G. (2001). Functional brain imaging based on ERD/ERS. *Vision Research*.

[53] Huppert T. J., Diamond S. G., Franceschini M. A., Boas D. A. (2009). HomER: a review of time-series analysis methods for near-infrared spectroscopy of the brain. *Applied Optics*.

[54] Cope M., Delpy D. T., Reynolds E. O., Wray S., Wyatt J., van der Zee P. (1988). Methods of Quantitating Cerebral Near Infrared Spectroscopy Data. *Oxygen Transport to Tissue X*.

[55] Guyon I., Elisseeff A. (2003). An introduction to variable and feature selection. *Journal of Machine Learning Research*.

[56] Braun M. L., Buhmann J. M., Müller K.-R. (2008). On relevant dimensions in kernel feature spaces. *Journal of Machine Learning Research*.

[57] Holm S. (1979). A simple sequentially rejective multiple test procedure. *Scandinavian Journal of Statistics*.

[58] Guyon I., Weston J., Barnhill S., Vapnik V. (2002). Gene selection for cancer classification using support vector machines. *Machine Learning*.

[59] Gupta R., Banville H. J., Falk T. H. PhySyQX: a database for physiological evaluation of synthesised speech quality-of-experience.

[60] Zacks J. M. (2008). Neuroimaging studies of mental rotation: A meta-analysis and review. *Journal of Cognitive Neuroscience*.

[61] Price C. J. (2010). The anatomy of language: a review of 100 fMRI studies published in 2009. *Annals of the New York Academy of Sciences*.

[62] Ischebeck A., Zamarian L., Siedentopf C. (2006). How specifically do we learn? Imaging the learning of multiplication and subtraction. *NeuroImage*.

[63] Fehr T., Code C., Herrmann M. (2007). Common brain regions underlying different arithmetic operations as revealed by conjunct fMRI-BOLD activation. *Brain Research*.

[64] Curtis C. E., D'Esposito M. (2003). Persistent activity in the prefrontal cortex during working memory. *Trends in Cognitive Sciences*.

[65] Kleber B., Birbaumer N., Veit R., Trevorrow T., Lotze M. (2007). Overt and imagined singing of an Italian aria. *NeuroImage*.

[66] Gunji A., Ishii R., Chau W., Kakigi R., Pantev C. (2007). Rhythmic brain activities related to singing in humans. *NeuroImage*.

[67] Ino T., Inoue Y., Kage M., Hirose S., Kimura T., Fukuyama H. (2002). Mental navigation in humans is processed in the anterior bank of the parieto-occipital sulcus. *Neuroscience Letters*.

[68] Pfurtscheller G., Neuper C. (1997). Motor imagery activates primary sensorimotor area in humans. *Neuroscience Letters*.

[69] Pfurtscheller G., Brunner C., Schlögl A., Lopes da Silva F. H. (2006). Mu rhythm (de)synchronization and EEG single-trial classification of different motor imagery tasks. *NeuroImage*.

[70] Ishai A., Haxby J. V., Ungerleider L. G. (2002). Visual imagery of famous faces: effects of memory and attention revealed by fMRI. *NeuroImage*.

